# Pausing controls branching between productive and non-productive pathways during initial transcription in bacteria

**DOI:** 10.1038/s41467-018-03902-9

**Published:** 2018-04-16

**Authors:** David Dulin, David L. V. Bauer, Anssi M. Malinen, Jacob J. W. Bakermans, Martin Kaller, Zakia Morichaud, Ivan Petushkov, Martin Depken, Konstantin Brodolin, Andrey Kulbachinskiy, Achillefs N. Kapanidis

**Affiliations:** 1 Biological Physics Research Group, Clarendon Laboratory, Department of Physics, University of Oxford, Parks Road, Oxford, OX1 3PU UK; 2 Junior Research Group 2, Interdisciplinary Center for Clinical Research, Friedrich-Alexander-University Erlangen-Nürnberg (FAU), Hartmannstrasse 14, 91052 Erlangen, Germany; 3 Department of Biochemistry, University of Turku, 20014 Turku, Finland; 4 Institut de Recherche en Infectiologie de Montpellier (IRIM) UMR9004 CNRS-Université de Montpellier, 1919 Route de Mende, 34293 Montpellier, France; 5 Institute of Molecular Genetics, Russian Academy of Sciences, Moscow, 123182 Russia; 6 Department of Bionanoscience, Kavli Institute of Nanoscience, Delft University of Technology, Van der Maasweg 9, 2629 HZ Delft, The Netherlands

## Abstract

Transcription in bacteria is controlled by multiple molecular mechanisms that precisely regulate gene expression. It has been recently shown that initial RNA synthesis by the bacterial RNA polymerase (RNAP) is interrupted by pauses; however, the pausing determinants and the relationship of pausing with productive and abortive RNA synthesis remain poorly understood. Using single-molecule FRET and biochemical analysis, here we show that the pause encountered by RNAP after the synthesis of a 6-nt RNA (ITC6) renders the promoter escape strongly dependent on the NTP concentration. Mechanistically, the paused ITC6 acts as a checkpoint that directs RNAP to one of three competing pathways: productive transcription, abortive RNA release, or a new unscrunching/scrunching pathway. The cyclic unscrunching/scrunching of the promoter generates a long-lived, RNA-bound paused state; the abortive RNA release and DNA unscrunching are thus not as tightly linked as previously thought. Finally, our new model couples the pausing with the abortive and productive outcomes of initial transcription.

## Abstract (web summary)

RNA synthesis by bacterial RNA polymerase is interrupted by pauses but their role in RNA synthesis is poorly understood. Here the authors use single-molecule FRET and biochemical analysis to show that pausing regulates branching between the abortive and productive outcomes of initial transcription.

## Introduction

Transcription initiation by DNA-dependent RNA polymerase (RNAP) constitutes the first and often decisive step in gene expression in bacteria. To balance the output of transcription with environmental and cellular needs, an extensive set of molecular mechanisms has evolved to regulate the efficiency and specificity of transcription initiation^1^. The regulatory mechanisms are either directly encoded in the transcribed DNA sequence or mediated by protein transcription factors or small-molecule signals. The target of transcription initiation regulators may be the function of RNAP itself, or the accessibility or affinity of promoters for RNAP. Further regulation occurs in the elongation and termination phases of transcription^2–5^.

**Fig. 1:**
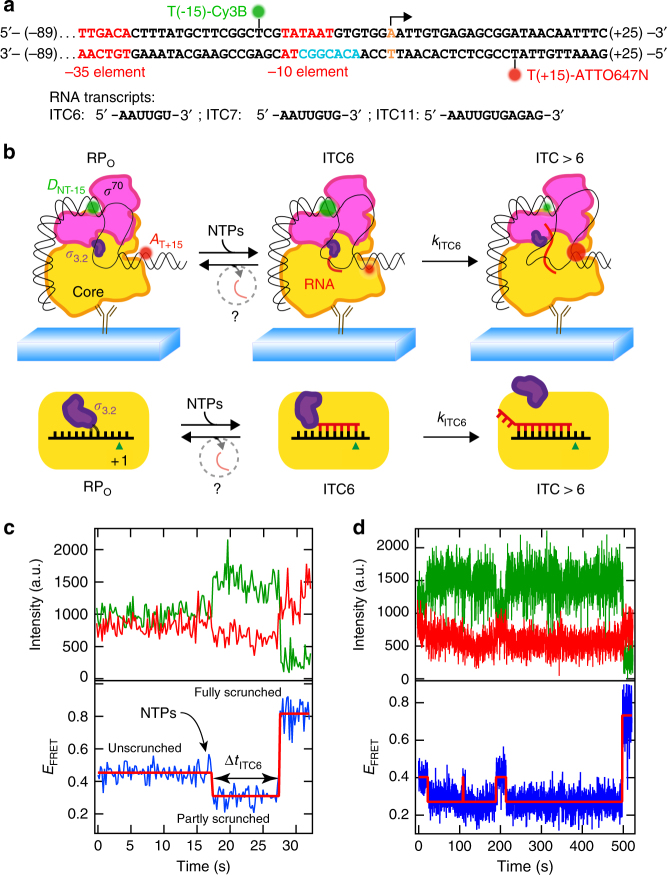
Initial transcription monitored at the single-molecule level. **a** Representation of the premelted (turquoise font) WT DNA promoter used in the single-molecule experiments (Supplementary Fig. 1a). The – 35 and – 10 elements are represented in red. The promoter was donor labeled at – 15 position (green sphere) of the non-template DNA strand and acceptor labeled in + 15 (red sphere) position of the template DNA strand. An arrow above the base in orange font indicates the + 1 position. All the promoters used in the study are described in Supplementary Fig. 1a. **b** Schematic of the initial transcription experiment (Methods). Above: using TIRFM-based smFRET, we monitored the E_FRET_ variations of the donor–acceptor pair upon NTP addition. The RNAP fluctuates between RP_O_, ITC6, and ITC > 6, to eventually escape the initiation phase toward the elongation phase, or to release the nascent RNA; below: cartoon that magnifies the interactions between the 5′-RNA end and *σ*_3.2_ and the position of the 5′-RNA end. **c** Fluctuations in the donor (green) and acceptor (red) dyes intensities (above) and the resulting E_FRET_ (below, blue), showing the variation of *E*_FRET_ from an Unscrunched (US) FRET state, followed by the Partly Scrunched (PS) FRET state upon NTP addition, and ending in the Fully Scrunched (FS) FRET state. Experimental conditions: 200 ms time points (100 ms ALEX, Methods), 500 µM ApA, and 80 µM All NTP. **d** Similar experiment conducted as described in **c**, with the RP failing multiple times before reaching the FS FRET state. Experimental conditions: 200 ms time points, 500 µM ApA, and 30 µM of all NTPs and WT promoter. The red solid lines in the lower panels in **c** and **d** represent the FRET states extracted from hidden Markov modeling (HMM) (Methods)

To perform promoter-specific transcription initiation, the bacterial RNAP core associates with housekeeping *σ*^70^ initiation factor (or one of the alternative *σ*-factors) to form an RNAP holoenzyme^6,7^. The holoenzyme employs sequence-specific interactions between the *σ*^70^ and the – 35 and – 10 promoter elements (Fig. 1a) to form an initial RNAP–DNA closed complex, and to isomerize to the catalytically competent RNAP–promoter open complex (RP_O_)^8,9^ (Fig. 1b). During initial RNA synthesis, strong interactions with the DNA hold the RNAP at the promoter, resulting in the build-up of “scrunching” of downstream DNA, a conformational change that increases the size of the DNA bubble^10–13^. The eventual break-up of RNAP–promoter contacts and the escape to elongation relax the scrunched DNA^11^. The productive promoter escape pathway competes with abortive initiation, an unproductive pathway wherein the short nascent RNA is thought to dissociate prematurely, resetting the initially transcribing complex (ITC) to RP_O_^11,14–18^. Although conformational strain resulting from the DNA scrunching may promote abortive initiation^11^, multiple other factors such as the presence of the *σ*_3.2_ region (which obstructs the entry to the RNA-exit channel^19–22^), strong RNAP–promoter interactions^9,16,23^, and the initially transcribed sequence^24,25^ also contribute.

The step that defines the overall rate of transcription initiation varies between promoters^9,16,23^. In many *σ*^70^-dependent promoters, the rate-limiting step is attributed to the half-life of RP_O_ or the rate of promoter escape. An extensively studied example of an escape-limited promoter is *lacUV5*^26^, which produces substantial amounts of abortive products; further, transcriptional pausing was identified in the ITC formed on *lac* promoter after the synthesis of 6-nt RNA, in part due to the clash of the 5′-RNA end with the *σ*_3.2_ region^27,28^.

Recent advances in structural characterization of bacterial transcription initiation complexes have created intriguing hypotheses on how specific molecular interactions and conformational changes drive holoenzyme formation, promoter recognition, open complex formation^29^, and initial RNA synthesis^12,20,30^. Complementing this structural insight with detailed functional analysis is hampered, however, by the multi-step, asynchronous nature of transcription initiation pathways. Single-molecule techniques, which can provide a direct readout for several mechanistic steps and resolve co-existing reaction pathways, are well positioned to overcome the complexity of transcription initiation^31–34^.

Here we combine single-molecule and biochemical analysis of initial transcription to explore the mechanistic basis of the pause encountered by ITC6 on a consensus variant of *lac* promoter, i.e., with consensus − 35 and − 10 elements, and a 17 bp spacer. We present evidence that the ITC6 pause represents a major control point where the ITCs branch to three competing downstream reaction pathways: pause exit by productive transcription; abortive-RNA release; and slow cycling between DNA conformations with different extents of scrunching but without RNA release. The partitioning between these three paths and their kinetics depends on distinct interactions and structural elements. The rate of productive pause exit is synergistically controlled by the initial transcribed sequence and the interaction of the 5′-RNA end with *σ*_3.2_ region, whereas perturbing RNAP interactions with the initially transcribed region favors the entry into the scrunching/unscrunching pathway.

## Results

### High-resolution observation of initial transcription

To monitor the kinetics of transcription initiation at the single-molecule level, we developed a Förster resonance energy transfer (FRET) sensor with fluorophore-labeled consensus *lac* promoter for real-time imaging of individual transcription-engaged RNAP–promoter complexes. Earlier studies of this consensus *lac* promoter derivative identified a strong pause at ITC6^27,35^. We modified the original promoter design in two ways to allow in-depth biophysical analysis of the rate-limiting ITC6 pause (Fig. 1a and Supplementary Fig. 1a). First, we extended the upstream region of the promoter from − 39 to − 89, to enhance RP_O_ formation and provide a more native DNA-length context for RNAP–DNA interactions^24,36^. Second, we moved the acceptor dye from position + 20 to + 15, to obtain distinct FRET signals for different steps of the initiation pathway. With this configuration, we clearly separated and calibrated (Methods) FRET readouts for three structural states through downstream DNA scrunching^10^: an unscrunched (US) open complex RP_O_ (*E*_FRET_ = 0.49 ± 0.003), a partly scrunched (PS) paused complex ITC6 (*E*_FRET_ = 0.37 ± 0.001), and a fully scrunched (FS) pause-cleared complex at ITC11 (*E*_FRET_ = 0.80 ± 0.002) (Fig. 1b and Supplementary Fig. 1).

Upon addition of NTPs to RP_O_ complexes (either a NTP subset sufficient to reach the ITC11 complex or all NTPs), the FRET signal showed an almost instant transition from the US to the PS state (Fig. 1c, Supplementary Fig. 1b and Supplementary Fig. 1c), suggesting that the transcription complexes synthesized 6-mer RNA and paused. After the pause, the ITCs split into two main populations: the first population comprised “productive” ITCs that resumed transcription and progressed from the PS to the FS state by synthesizing an 11-mer (Fig. 1c). The second population comprised ITCs that returned from the PS to the US state (Fig. 1d); notably, such complexes could cycle multiple times (e.g., at ~ 100 s and ~ 200 s in Fig. 1d) between PS and US states until they eventually reached the FS state (e.g., at ~ 500 s in Fig. 1d).

### Determination of the lifetime of the ITC6 pause

Two elements appear to contribute to RNAP pausing at ITC6: (i) the clash of 5′-RNA end with the *σ*_3.2_ region (Fig. 1b), which blocks entry to the RNA-exit channel of RNAP^27^, and (ii) a specific sequence motif (a non-template Y^+6^G^+7^ in the transcribed DNA strand^35^) akin to that causing sequence-specific pausing in elongation^37–39^. We dissected the contributions of these two elements to the ITC6 pause using our single-molecule FRET assay.

To explore the steric-clash hypothesis, we modified the 5′-RNA end of the nascent transcript (and thus its interaction with *σ*_3.2_) by initiating transcription either using a synthetic dinucleotide (ApA) or using ATP, which adds a 5′-triphosphate tail to the 5′-RNA end. To evaluate the effect of the pause sequence motif on the detailed dynamics of initial transcription, we replaced the sequence T^+6^G^+7^ (on non-template DNA) with G^+6^T^+7^, creating a “ΔP promoter” (Supplementary Fig. 1a) that increased the overall rate of initial transcription by shortening the ITC6 pause^35^. In all experiments, the initiating ATP or ApA were held at 500 µM, a level significantly above the *K*_M_ of the RP_O_ for initiating nucleotides and dinucleotide primers^22^; we also varied the concentration of remaining NTPs (1–500 µM).

**Fig. 2:**
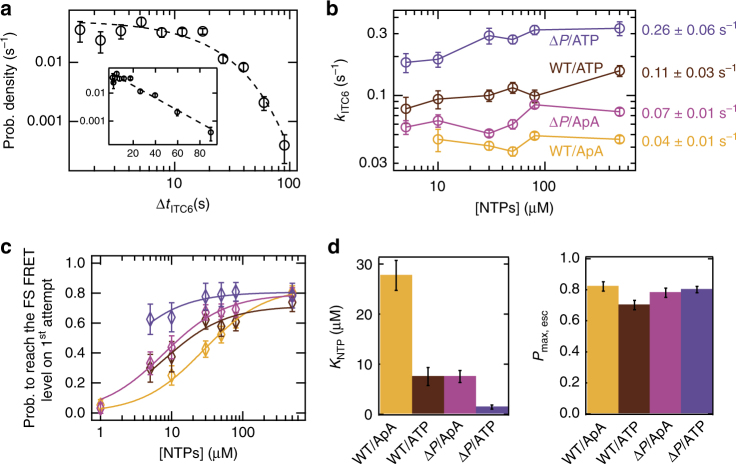
The pause characteristics are a function of the downstream DNA promoter sequence at the + 6 position and of the 5′-RNA end nature. **a** Probability density distribution for the Δ*t*_ITC6_ for the RP complexes behaving as in Fig. 1c. The dashed line is a single-exponential fit from a MLE. Inset: Log-lin representation of the same data. Experimental conditions: 500 µM ApA starting substrate, 80 µM all NTPs. **b** Δ*t*_ITC6_ lifetime extracted from single exponential MLE fit similar to **a** for different promoter/starting substrate conditions (as indicated in the panel), different NTP conditions, i.e., all NTPs for WT/ApA (yellow) and ITC11 (Supplementary Fig. 1a) for all others, and different NTP concentrations (Supplementary Table 1). In the ATP-initiated reactions, we did not use NTP concentration below 5 µM to prevent potential misincorporations of ATP (used at 500 µM for initiation purposes)^69^. On the right-hand side is indicated the mean ± SD of *k*_ITC6_ for each promoter/starting substrate condition. **c** Probability to reach the fully scrunched (FS) FRET level in a single attempt (Fig. 1c). The solid lines are fits to a binding isotherm of the form $p\left( \mathrm{{NTP}} \right) = P_{\rm max,esc} \times \left[ \mathrm{{NTP}} \right]/(\left[ \mathrm{{NTP}} \right] + K_{\mathrm{{NTP}}})$. The error bars are 95% confidence intervals. **d** *K*_NTP_ and *P*_max,esc_ extracted from **c**. Error bars are 1 SD extracted from the fit

We first analyzed the effects of the pause elements on the pause duration at ITC6 (Δ*t*_ITC6_) by focusing on the subpopulation of molecules displaying the US→PS→FS scrunching sequence (as in Fig. 1c). The dwell-time distribution for the ITC6 pause was well described by a single exponential (Fig. 2a; see Methods for analysis procedure), where the exit rate *k*_ITC6_ was ~ 1.5-fold lower for the wild-type (WT) promoter compared with the ΔP promoter using ApA (Fig. 2b). When we replaced ApA with ATP as the starting substrate and employed the remaining NTPs at above 30 µM, the ITC6 pause exit rate increased from 0.07 ± 0.01 to 0.26 ± 0.06 s^−1^ for the ΔP promoter and from 0.04 ± 0.01 to 0.11 ± 0.03 s^−1^ for the WT promoter, i.e., 2.5-fold enhancement in *k*_ITC6_ in the absence of the pause motif (Fig. 2b). These experiments demonstrate that the ITC6 pause duration is controlled both by the transcribed sequence and by the structure of the RNA 5′-end, which interacts with *σ*_3.2_.

We also noted that the NTP concentration did not influence significantly the *k*_ITC6_ (no more than 1.5-fold) for the WT promoter with either ApA or ATP as the starting substrate, or for the ΔP promoter with ApA as the starting substrate (Fig. 2b). The rate-limiting step in all these cases is thus neither the intrinsic catalytic activity of the transcription complex nor the binding of the incoming NTP substrate.

We next characterized the probability to exit the ITC6 pause on the first attempt (Fig. 2c). For this purpose, we counted the probability of ITCs to proceed via the reaction path depicted in Fig. 1c (single-scrunch pathway), with or without a detectable ITC6 pause, versus the path in Fig. 1d (cyclic scrunching/unscrunching pathway). ATP-initiated ΔP promoter complexes (Fig. 2c) exited the pause on the first attempt at higher probability at all tested (5–500 µM) NTP concentration compared with the other starting substrate-promoter conditions. The pause-exit probability for the ApA-initiated ΔP promoter, and the ATP- or ApA-initiated WT promoter decreased steeply from 0.8 toward 0 at low NTP concentrations (Fig. 2c). By fitting the probability *p*(NTP) to exit the ITC6 pause on the first attempt with a descriptive model similar to a binding isotherm (Fig. 2c), we extracted an apparent binding constant *K*_NTP_ and a maximal pause-exit probability *P*_max,esc_ (Fig. 2d). Overall, the WT promoter had a higher *K*_NTP_ compared with Δ*P* promoter complexes (28 ± 3 vs. 8 ± 1 µM, ApA), whereas ATP-initiated complexes had a lower *K*_NTP_ compared with ApA-initiated ones (8 ± 2 vs. 28 ± 3 µM, WT promoter; 1.7 ± 0.4 vs. 8 ± 2 µM, Δ*P* promoter). The probability *P*_max,esc_ was relatively constant, with 79 ± 5% of the molecules reaching the FS FRET level on the first attempt at saturating NTP concentration. These results suggest that ITCs can exit a weak ITC6 pause (Δ*P* promoter) efficiently even at low NTP concentration, while overcoming a strong ITC6 pause (WT promoter + ApA at the 5′-RNA end) requires higher NTP concentration (Fig. 2c). Interestingly, we observed that 98 ± 2% and 89 ± 3% of the complexes paused at ITC6 on WT promoter and Δ*P* promoter, respectively (averaged percentages for all NTP concentrations and ATP starting substrate), indicating that T^+6^G^+7^ motif enforces pausing at ITC6 (Supplementary Fig. 2b and Supplementary Note 1).

Finally, a fully double-stranded promoter (dsWT, Supplementary Fig. 1a) did not modify the ITC6 pause exit rate both for ApA and ATP starting substrates (Supplementary Fig. 2c), whereas the probability to reach the FS state during the first attempt on this promoter was also strongly decreased in the absence of a 5′-RNA end triphosphate (14 ± 4% vs. 58 ± 5%, Supplementary Fig. 2d), suggesting again that the 5′-RNA end triphosphate assists in the ITC6 pause exit.

### Structural determinants of transcription pathway partitioning

**Fig. 3:**
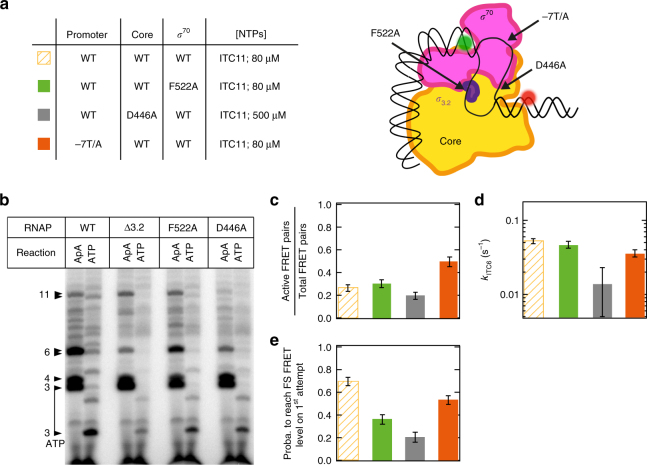
Core and *σ*^70^ mutants affect ITC6 pause exit probability. **a** Experimental conditions studied here and schematic of the different RP complex variants, all with ApA starting substrate. The same experiments have been performed with ATP starting substrate and are presented in Supplementary Fig. 2e, f, g. **b** Bulk reaction performed with a consensus *lac* promoter (Supplementary Fig. 1a) and different holoenzyme mutants, in ITC11 NTP conditions (500 µM ApA and 80 µM ATP/UTP/GTP or 500 µM ATP and 80 µM UTP/GTP) and 1 min incubation at 37 °C (the experimental procedure is described in the Supplementary Information). The band assignment is indicated on the left hand side of the gel. **c** The fraction of transcriptionally active RPs, which displayed NTP-dependent *E*_FRET_ changes, of all surface-immobilized RPs (see also, Methods: FRET pair localization and detection), in the experimental conditions described in **a**. **d** ITC6 pause exit rate for the experimental conditions described in **a**. **e** Probability to reach the FS FRET level on the first attempt for the experimental conditions described in **a**. Error bars are either 1 SD from 1000 bootstraps procedure **d** or 95% confidence interval **c**, **e**

Our single-molecule reaction trajectories demonstrated (Fig. 1) that the transcription complexes paused at ITC6 may either resume RNA extension or cycle between differently scrunched paused states. To establish the interactions contributing to the pathway partitioning, we engineered structural changes (Fig. 3a) in RNAP, *σ*^70^, and nucleic acids, and characterized the impacts on the function of ITCs.

We first explored the significance of *σ*_3.2_-template-strand DNA interaction by using F522A substituted *σ*^70^, which is deficient in an interaction between the – 4 template DNA base and *σ*_3.2_^29^, and affects initial transcription^22^. During transcription initiation, the F522A substitution decreased the amounts of ≤ 6 nt RNA products, likely because it decreased the physical barrier from their clash with the *σ*_3.2_ region^22^, but had little effect on the ITC11 formation (Fig. 3b). In the FRET assay, the F522A *σ*^70^ derivative retained similar activity (Fig. 3c and Supplementary Fig. 2e) and *k*_ITC6_ as the WT (Fig. 3d and Supplementary Fig. 2f). Instead, the substitution significantly decreased the fraction of complexes exiting the pause on the first attempt (70 ± 4% to 37 ± 4% for ApA starting substrate), independently of the use of ApA (Fig. 3e) or ATP (Supplementary Fig. 2g) as the starting substrate. The weakening of *σ*_3.2_ interaction with the template-strand DNA thus destabilizes the PS promoter conformation and biases the paused ITC6 toward the scrunching/unscrunching pathway. In comparison, the *σ*_3.2_ region deletion has a much more severe defects in transcription initiation and strictly requires dinucleotide primers for 11-mer synthesis^22,27^(Fig. 3b).

We next destabilized by βD446A substitution the binding of non-template guanine in the “CRE-pocket” (Core Recognition Element-pocket) of RNAP^29,37^. Our results align with an earlier observation of the CRE-pocket being involved in open complex formation and transcription start-site selection^40^; we observed three-fold less active RNAP–promoter complexes (ApA starting substrate, Supplementary Fig. 2e), which is only partly recovered when increasing the NTP concentration to 500 µM (Fig. 3c). We also observed, similar to the consensus elongation pause^37^, a reduced escape rate (0.10 ± 0.01 vs. 0.06 ± 0.01 s^−1^) from the ITC6 pause (Supplementary Fig. 2f). Both pauses are characterized by the presence of Y^−1^G^+1^ ntDNA motif. Our third observation reveals a potentially novel function for the CRE-pocket in helping to maintain the scrunched promoter DNA conformation; fourfold less (20 ± 5% vs. 70 ± 4%) complexes managed to escape the pause on the first attempt (Fig. 3e). In bulk transcription reactions, only a minor fraction of complexes formed by the D446A RNAP could extend RNA beyond six nucleotides, suggesting that the contacts of D446 with + 7G are critically important to exit the paused state (Fig. 3b)^35^.

To probe the effects of weakened interactions between *σ* region 2 and the – 10 promoter element, we replaced the consensus – 7 thymine in the non-template DNA by an adenine (– 7T/A, Supplementary Fig. 1a). The – 7 thymine is inserted into a pocket of σ in RP_O_^29,41,42^. However, as – 7T/A substitution resulted in only small changes in the ITC6 pause exit rate and the fraction of complexes exiting the pause on the first attempt (Fig. 3c, d), – 7T–*σ* interaction appears to have a minor role after RNA length is ≥ 6.

### Kinetics of promoter unscrunching/scrunching

**Fig. 4:**
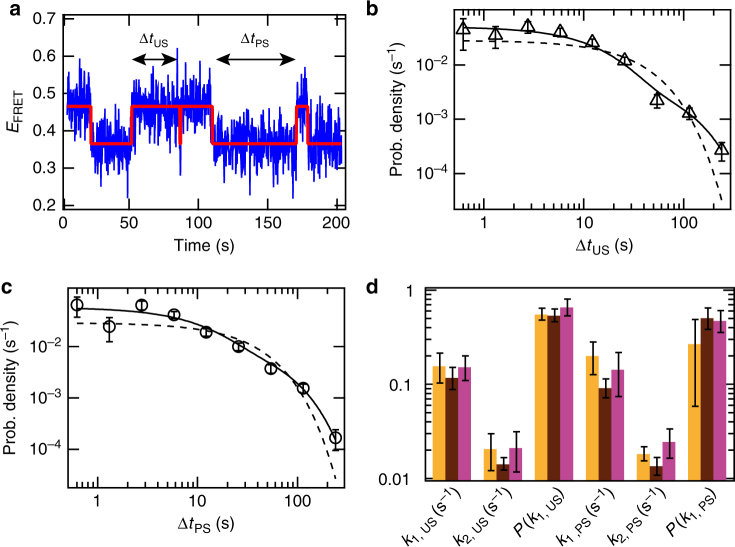
RP complexes that do not reach the FS FRET level alternate between US and PS FRET levels for long period of time. **a** Typical *E*_FRET_ trace where the RP alternates between unscrunched (US) and partly scrunched (PS) DNA promoter FRET levels. The red solid line represents the FRET levels extracted from empirical Bayesian probability hidden Markov model. We collect the dwell times Δt for each FRET level. Experimental condition: WT DNA promoter; NTP start: ApA; NTP: U/G/ATP 80 µM. **b**, **c** Probability density distribution of the dwell times Δ*t*_US_ and Δ*t*_PS_, respectively, for the experimental conditions described in **a**, with its single and double exponential MLE fit (dashed and solid line, respectively, Methods for MLE fit procedure). **d** Average for all the NTP concentration (Supplementary Fig. 2e-g) of the double exponential MLE parameters (*k*_1_ and *k*_2_ exit rates, and *P* probability of being in the exponential with *k*_1_ exit rate) for US and PS FRET states averaged over all NTP concentrations, for the conditions described in Fig. 2a and Supplementary Table 1. Color scheme as in Fig. 2

We next analyzed the molecules whose pausing at ITC6 was followed by cyclic unscrunching/scrunching events. These molecules may cycle for tens to hundreds of seconds between the PS and US states until they reach the FS FRET level or the dyes bleached (Figs. 1d and 4a). For this cycling population, we generated probability density distributions for the dwell times in PS (Δ*t*_PS_) and US (Δ*t*_US_) states (Fig. 4b, c). Both PS and US distributions showed a similar trend, with dwell times varying from ~ 0.4 s to ~200 s. The distributions were fitted well by a two-exponential probability distribution (solid lines, Fig. 4b, c; dashed lines depict a single-exponential function) (Methods). The fit thus defines for the US and PS states the exit rates *k*_1_ and *k*_2_, as well as the probability *P*(*k*_1_) to exit a state with rate *k*_1_ (Supplementary Fig. 2e, g).

We applied this data analysis to WT promoter reactions initiated with ApA or ATP, and the Δ*P* promoter initiated with ApA (Fig. 2b). We did not include ATP-initiated ΔP promoter results because most complexes exited the ITC6 pause directly to the FS state (Fig. 2c). We first noted that the exit rates *k*_1_ and *k*_2_, as well as the *P*(*k*_1_) probabilities of PS and US states, remained fairly constant in all used NTP concentrations (Supplementary Fig. 2h, j). We observed a single exception with the ITC on the ApA-initiated WT promoter, which showed a decreased probability *P*(*k*_1, PS_) at higher NTP concentrations (right panel, Supplementary Fig. 2h). We observed, in average that the US and PS states had nearly identical kinetic parameters: *k*_1_ ~ 0.15 s^−1^, *k*_2_ ~ 0.02 s^−1^, and *P*(*k*_1_) ~ 0.6. Notably, these values were also independent of the NTP subset used (allowing maximal transcript lengths 7 or 11), the nature of the RNA 5′-end, or any of the tested RNAP-promoter variations (Fig. 4d and Supplementary Fig. 2k, l, m).

The remarkable insensitivity of the unscrunching/scrunching kinetics to the tested parameters, and in particular to the NTP concentration, strongly suggests that the complexes entering the unscrunching/scrunching pathway are catalytically inactive. Even though the complexes are ultimately able to resume RNA synthesis, the unscrunching/scrunching pathway delays the promoter clearance by tens of seconds (Fig. 1d).

### Promoter unscrunching and RNA release

**Fig. 5:**
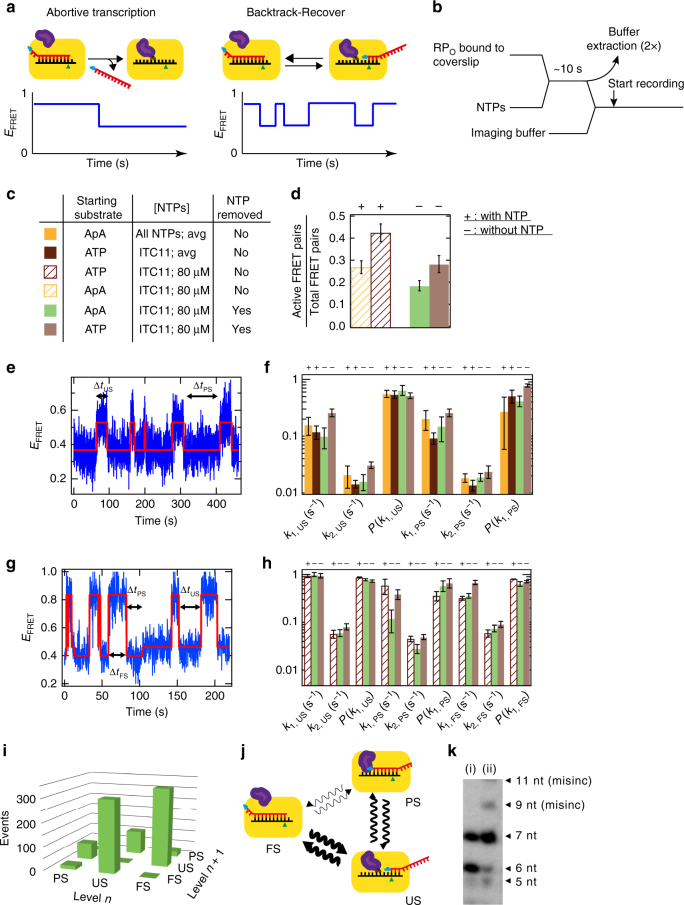
Post-RNA synthesis DNA promoter scrunching kinetics are independent of the presence of NTP. **a** Schematic of the RPs possible behavior and the corresponding FRET signal. **b** Schematic of the experiment. **c** Table presenting the experimental variables probed in Fig. 5. Note that the data represented in brown and yellow are from Fig. 2 and are averaged (avg) values for all the concentration of NTP probed in these experiments. Here, we used the WT DNA promoter (Supplementary Fig. 1) and wild-type holoenzyme (Methods). **d** Ratio of the numbers of active FRET pairs, i.e., that display scrunching/unscrunching cycles, over the total number of FRET pairs after selection (Methods: FRET pair localization and detection). The + and – above the bars indicate the presence and the absence, respectively, of NTPs during the experiment. An identical notation is used in **f** and **h**. **e** Experimental FRET trace after NTP removal **b** showing an RP complex alternating between US and PS, but not in the FS FRET level. The experimental conditions used for the acquisition of this trace correspond to the light green color code in **c**. **f** Scrunching kinetics (*k*_1_, *k*_2_, and *P*(*k*_1_)) extracted from a double-exponential MLE fit analysis of the Δ*t*_US_ and Δ*t*_PS_ distributions from the traces alike **e**. **g** Experimental FRET trace after NTP removal (as in **b**) showing an RP complex alternating between US, PS and FS FRET level. **h** Scrunching kinetics (*k*_1_, *k*_2_, and *P*(*k*_1_)) extracted from a double-exponential MLE fit analysis of the Δ*t*_US_, Δ*t*_PS_, and Δ*t*_FS_ distributions from the traces alike **g** for the conditions described in **c**. **i** 3D histogram showing the number of transition between two different FRET levels for two consecutive dwell times *n* and *n* + 1 for the traces acquired as described in **c**. **j** Schematic representing the results of **i**; we use wavy arrows to highlight that these are complex transitions involving two timescales; the thicker the arrow, the more likely the transition. **k** Transcript retention experiment using magnetic-bead-attached RP complexes incubated in ITC7 conditions and rinsed (i), then restarted after 15 s by chasing 80 µM GTP (ii) (Supplementary Protocol in Supplementary Methods). The 9mer and 11mer originated from GTP misincorporations

The discovery of extensive unscrunching/scrunching cycling raises intriguing questions about its relationship with abortive initiation. Is the nascent RNA released or retained in unscrunching event, and is the subsequent re-scrunching driven by the synthesis of a new RNA (Fig. 5a)? To address the questions, we devised a three-step assay (Fig. 5b) in several conditions (Fig. 5c): (i) RNA synthesis was triggered for ~ 10 s with ITC11 (Fig. 5e, g) or ITC7 (Supplementary Fig. 3a) NTP subsets, (ii) the surface was extensively rinsed to remove NTPs, (iii) surface-bound complexes were re-imaged. To our surprise, 28 ± 4% (ApA, ITC11) and 18 ± 2% (ATP, ITC11) of initially active complexes cyclically  unscrunched/scrunched also in the absence of NTPs (Fig. 5d). The fraction of unscrunching/scrunching molecules was comparable to those observed in the continuous presence of NTPs (27 ± 3%, ApA starting substrate; 42 ± 4% ATP) (Fig. 5d). Scrunching/unscrunching by the NTP-depleted complexes lasted for hundreds of seconds, being only limited by dye bleaching (Fig. 5e, g). Consistent with the maximal RNA length, complexes pulsed with ITC7 NTPs sampled only US and PS states (Supplementary Fig. 3a), whereas complexes pulsed with ITC11 NTPs could additionally occupy the FS state (Fig. 5g). This confirms that we did not observe re-synthesis of a 11mer transcript, as we observed earlier that the complex could not escape the ITC6 pause at low NTP concentration (Fig. 2c). Our results clearly demonstrate that the extended cycling in different scrunching states does thus not require active RNA synthesis.

Analysis of the complexes pulsed with ITC11 NTPs identified two types of unscrunching/scrunching molecules: the first cycled between US and PS FRET levels only (Fig. 5e), whereas the second cycled between US, PS, and FS FRET levels (Fig. 5g). The US/PS subpopulation included 48 ± 6% (ApA starting substrate) or 39 ± 5% (ATP starting substrate) of all cycling molecules, respectively (Supplementary Table 1). The US/PS and US/PS/FS subpopulations likely represent the ITCs, which at the moment of NTP withdrawal had synthesized 6- and 11-nt RNAs, respectively. The US/PS subpopulation showed similar unscrunching/scrunching kinetics to what was observed with NTPs, i.e., *k*_1_ = 0.16 ± 0.07 s^−1^, *k*_2_ = 0.02 ± 0.004 s^−1^, and *P*(*k*_1_) = 0.57 ± 0.05 (Fig. 5f). The US/PS/FS subpopulation instead sampled all scrunching states almost an order of magnitude faster compared to the US/PS subpopulation (i.e., *k*_1_ = 0.96 ± 0.04 s^−1^, *k*_2_ = 0.07 ± 0.01 s^−1^, and *P*(*k*_1_) = 0.79 ± 0.07 for US, Fig. 5h), independently of using ATP or ApA for initiation (Fig. 5f, h).

Close inspection of the trajectories belonging to the US/PS/FS subpopulation revealed that the two most frequently encountered state transitions were FS→US and its reversal US→FS (Fig. 5i and Supplementary Fig. 3f); this was also the case in the continuous presence of NTP (Supplementary Fig. 3g). The US→PS and PS→US transitions were about 4-fold less frequent, whereas PS→FS or FS→PS transitions were only rarely observed. This data clearly indicate that RPs engaged in the unscrunching/scrunching pathway do not share the same linear US→PS→FS reaction coordinate of ITCs engaged in productive transcription (Fig. 5j). We also note the absence of any temporal correlation between two successive state dwell times (dt_*n*_ and dt_*n* + 1_), independently of the scrunching state they originate from (right hand side, Supplementary Fig. 3b, c, d), supporting a memory-less transition from one state to the next.

### Paused ITC may undergo abortive initiation or hold RNA

Our FRET assay monitors the conformation of the promoter DNA and thus does not provide a direct readout for the presence of RNA in the ITCs. As pulsed RNA synthesis generated ITCs that cycle for several min between scrunched states, we assumed that these ITCs retain the nascent RNA in the transcription bubble. The assumption generates two testable hypotheses: first, RNA is slowly released from NTP-deprived ITCs; second, RNAs retained in ITCs are extendable upon NTP reintroduction.

To determine the profile and time dependence of RNA release from ITCs, we immobilized biotinylated RP_O_ complexes to streptavidin-coated magnetic beads. The complexes were pulsed for 10 s with the ITC7 NTP subset (containing α^32^P-UTP), pulled down, washed, and immersed into NTP-free reaction buffer; beads and supernatant were then analyzed at specified times to obtain the time-dependent profile of retained and released RNAs (Supplementary Fig. 4a, b). Our results showed that the RNA-release kinetics was strikingly biphasic: many ITCs released their RNA within the first 2 min, the release being almost quantitative for the shortest RNAs (~ 95% of 3–4-mers) and less efficient for 5-, 6-, and 7-nt RNAs (45%, 80%, and 80%, respectively; Supplementary Fig. 4b). After the rapid initial phase, the amount of released 6- or 7-nt RNA increased only marginally. After 15 min, still ~ 20% of 6–7-nt RNA remained bound in the ITCs. This amount is two-fold lower than what we measured in similar NTP-pulsed single-molecule experiments, where most of the active ITCs were sampling the unscrunching/scrunching states for several min (Fig. 5d). We also tested the retention of short RNAs in transcription complexes formed under the ITC11 conditions and found that comparable amounts of 6-nt transcripts remained bound to RNAP even after prolonged incubation of the complexes in the absence of NTPs (Supplementary Fig. 4c). This is in agreement with the PS and FS FRET states observed in the absence of NTPs (Fig. 5e, g).

To probe whether the stalled ITCs retaining 6-nt RNA for an extended period of time can resume active transcription, we chased the immobilized and washed ITCs with the next incoming nucleotide (GTP). We observed that the 6-nt RNA became converted quantitatively to 7-nt RNA (Fig. 5k; longer products appear due to mis-incorporation), indicating that the ITCs both retain the nascent RNA in the transcription bubble, and can access the catalytically active conformation.

In summary, the biochemical analysis revealed two populations of stalled ITCs: 70–80% of ITCs that enter the abortive initiation pathway (rapidly releasing the nascent RNAs) and 20–30% of ITCs that retain 6–7-nt RNA products and catalytic competence for tens of min after NTP depletion. These results clearly show that the nascent RNA can be stably trapped within the cyclically unscrunching/scrunching RP complex, until being eventually elongated.

## Discussion

In this study, we employed a refined, high contrast single-molecule FRET assay to quantitatively dissect the reaction pathway and kinetics of the ITCs on the consensus *lac* promoter. We specifically examined the role of the *σ*_3.2_ region, the nature of pausing, and pausing-related conformational changes such as scrunching/unscrunching in the presence and absence of RNA release.

**Fig. 6:**
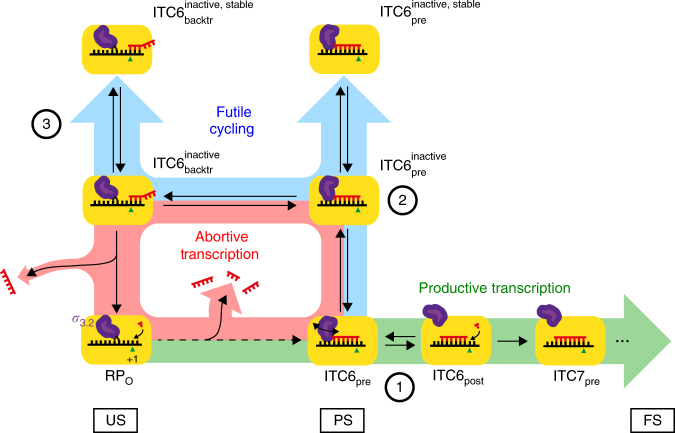
Model for initial bacterial transcription. The progress of initial transcription is illustrated by depicting RNAP (yellow block) at key points of the inferred mechanism. The mechanism includes three competing reaction pathways, which the ITC can embark on. Productive Transcription pathway (highlighted in green) results in promoter escape and synthesis of full-length RNA. Abortive Initiation pathway (highlighted in red) leads to the synthesis and dissociation of short RNA products. Futile Cycling (highlighted in blue) temporarily traps the ITC6 into catalytically inactive interconverting pre-translocated and backtracked states, respectively. Purple finger shows the different conformations of *σ*_3.2_. Green triangle marks the template base for the next incoming nucleotide in the active site of RNAP. Red and black strands represent the nascent RNA and template DNA, respectively. The multi-timescale transitions between PS and US in Fig. 5j have here been broken up to suggest a simple composition that would give rise to the two timescales observed in Fig. 4b, c. The numeration (1, 2, and 3) indicates the three significant molecular mechanisms described by the model: the initial barrier imposed by *σ*_3.2_ to the transcript elongation, the subsequent loss of catalytic conformation and the RNA-dependent reversible backtracking, respectively. The US, PS, and FS FRET levels observed during the experiments are indicated at the bottom of the schematic

The *σ*_3.2_ region has been described as a good candidate for causing pausing, and structural, biochemical and single-molecule biophysical studies have confirmed that by occluding the RNA-exit channel of RNAP the *σ*_3.2_ region forms a barrier for the elongation of the nascent transcript past 5–6 nt^12,19,20,27^ (Figs. 1b and 6). Consistent with these results, we recently showed that partial deletion of *σ*_3.2_ significantly diminished pausing at ITC6^27^. However, since the same *σ*_3.2_ derivative was associated with accelerated conformational dynamics in the open complex^43^, a possibility existed that some of the inferred effects on pause kinetics were indirect (e.g., due to instability of the template strand conformation in the DNA binding cleft leading to increased abortive RNA release and shortening of the ITC6 pause). Our new finding that the triphosphate moiety at the 5′-RNA end, which specifically interacts with the *σ*_3.2_^12,20^, both shortens the half-life of ITC6 pause and increases the probability of productive pause exit further suggests that *σ*_3.2_ is a major pause determinant in initial transcription. We also demonstrated that substitution of the *σ*_3.2_ residue F522, which contacts template DNA upstream of the active site and represents a barrier for initial RNA synthesis^22,29^, decreases the probability of successful RNA extension beyond the PS state. Thus, competition between the RNA 5′-end and residues from the *σ*_3.2_ region is required for efficient promoter escape, likely by promoting *σ*_3.2_ extrusion.

The 80–90% probability to enter the pause may reflect the presence of transcriptionally non-permissive (pausing RPs) and permissive (non-pausing RPs) *σ*_3.2_ conformations present in different ITC6 complexes. Based on the structural considerations^12,20^ and the stage of initial transcription (ref. ^27^; this study), the clash between *σ*_3.2_ and RNA 5′-end may hamper the movement of the template DNA and/or RNA to the post-translocated register, and therefore stabilize the pre-translocated state^27,35^. We provide an additional evidence in favor of this hypothesis by showing that βD446A RNAP (which de-stabilizes the post-translocated^37^) displayed twofold decreased ITC6 pause exit rate. However, because the pause exit rate did not strongly depend on the NTP concentration, the pause is not directly controlled by the thermodynamic equilibrium between the pre- and post-translocated states of ITC6. By similar reasoning, the pause is also not controlled by the catalytic rate of post-translocated ITC6. We thus postulate that the pause-controlling step is kinetic and involves relatively slow repositioning of the *σ*_3.2_ tip in a way that the barrier to forward translocation is removed. The highest observed pause exit rate on the promoter variant lacking the consensus pause motif (0.3 s^−1^ for the ΔP promoter, Fig. 2b) may reflect the rate of ITC6 pre→post translocation that we suggest to be controlled by *σ*_3.2_ repositioning, increasing dramatically the lifetime of the pre-translocated state in comparison to transcription elongation^44^ (~ 265-fold: 2.3 s vs. 8.7 ms). Importantly, several studies of pausing during transcription elongation have shown the predisposition of the pre-translocated RNAP to isomerize into a catalytically inactive off-pathway state, known as the elemental pause^2,37,38,45^. The *σ*_3.2_-dependent translocation barrier encountered during initial transcription may thus act, by accumulating the pre-translocated ITC6, to increase the probability to isomerize into an elemental pause-like state (Fig. 6).

We further noticed that the pyrimidine/guanine [(Y)/G] motif, first identified in elongation consensus pauses^37,38^, also affects pausing during transcription initiation (ref. ^35^ and this study). According to our current data the pause exit rate during initiation (~ 0.3 s^−1^) is similar to that during elongation (~ 0.5 s^−1^)^38^. The substitution of the motif increased both the pause exit rate and the probability to exit the pause. In contrast, substitution D446A in the CRE-pocket, which was previously shown to increase consensus pausing^37^, also impaired RNA extension in the ITC6. Overall, it appears that the first events leading to a pause during initiation and elongation phases of transcription are similar: an energetic (transcribed sequence in elongation) or physical (*σ*_3.2_ in initial transcription) barrier to translocation delays RNAP in the pre-translocated register^35^ from where the protein can, with sequence-dependent efficiency, branch-off to a catalytically inactive elemental pause state (Fig. 6).

Although the entry of ITC6 into the elemental pause was nearly obligatory (80–90% of trajectories showed the pause, Supplementary Fig. 2b), a significant fraction (~ 20% at saturating NTP concentration, Fig. 2d) of the RNAP complexes did not exit this pause on the first attempt, but instead embarked on another reaction pathway involving cyclic unscrunching/scrunching events. Unscrunching mechanistically resembles backtracking and leads to the displacement of 3′-RNA end from the pre-translocated register (predominant in PS state) towards the NTP-entry channel in US state. The net effect is a long-duration catalytic inactivation of ITC6 (Fig. 4). Interestingly, though promoter scrunching has been associated with intermediate stressed states, we did not observe a different lifetime for US, PS, and FS states (Fig. 5h), suggesting that the transitions connecting the scrunch states are not dominated energetically by the possible intermediate stressed states. Backtracking during initial transcription was also observed using magnetic tweezers^28^ (see Supplementary Discussion). The probability to enter the unscrunching/scrunching pathway inversely correlated with NTP concentration (Fig. 2c); at periods of low cellular NTP pool, the unscrunching/scrunching mechanism may thus efficiently inhibit promoter escape and transcript levels. Furthermore, perturbation of RNAP interactions with the DNA template and RNA transcript, e.g., by *σ*_3.2_-F522A or βD446A substitutions, favored the partitioning of ITC6 into the unscrunching/scrunching pathway (Fig. 3d and Supplementary Fig. 2g). This finding may further imply that native promoter and initially transcribed sequences encode efficient promoter-escape kinetics because they disfavor ITC partitioning into the non-productive unscrunching/scrunching pathway. Consistently, Record and colleagues^46^ recently reported the correlation of stronger holoenzyme–discriminator (promoter sequence between the – 10 element and transcription start site) interaction with the production of longer abortive RNAs, while producing a higher yield of promoter escape.

Previous single-molecule studies assumed a direct link between unscrunching and abortive transcription^11,17,27,28^. However, those studies focused on the DNA conformation and did not evaluate the retention of RNA in the transcription complexes. Our data demonstrated that brief pulsing of open complexes with NTPs resulted in a population of ITCs that kept on cycling between US/PS/FS states for an extended period of time (Fig. 5), and that the transcripts could remain stably attached to the ITC for at least 45 min (Supplementary Fig. 4). Backtracking to the US state shortens the template DNA–RNA hybrid to ≤ 5 bp, and should reduce the hybrid lifetime. However, the possibility of a short hybrid that locks the transcript into the DNA-binding cleft is supported by the observation of a 4-nt RNA bound to a bacterial open promoter complex in crystals^41^. Furthermore, the backtracked RNA potentially forms interactions in the NTP-entry channel, as observed in the yeast RNAP II^47^. Taken together, the stability of the short hybrid within the complex and the positive interactions between the RNA and the NTP entrance channel may support transcript retention upon promoter unscrunching.

Recent work^46^ has also noted that RP_O_ complexes on λP_R_ and T7A1 promoters were divided into two populations upon NTP addition: a first population (30–45% of all complexes) that rapidly (within 10 sec) synthesized long RNA (ITC > 10) and a second population that was stalled in early transcription (ITC < 10), and that released RNA slowly, similarly to moribund complexes^48^. We propose that these two populations, i.e., the population producing quickly long RNAs and the moribund complexes, are consistent with the two populations we described here, i.e., the RP complexes that exited the ITC6 pause on the first attempt (Fig. 1c), and the population that entered the cyclic unscrunching/scrunching state from the ITC6 pause (Fig. 1d), respectively (Supplementary Discussion).

Our new findings are summarized in a kinetic model of the transition to productive transcription (Fig. 6 and Supplementary Discussion).

The dependence of entry and recovery from the pause states implies wide variation in the kinetics of initial transcription across the bacterial promoter sequence space. On the consensus *lac* and similar promoters, the molecular mechanism of pausing sensitizes the efficiency of promoter escape to NTP concentration, potentially trapping the RNAP to the promoter in a “ready-to-fire” or “poised” mode until improved growth conditions lead to the replenishing of cellular NTP pool^49–51^. The trapping of poised RNAPs at or near the promoter thus emerges as a common transcription regulation strategy achievable by different molecular mechanisms. For example, the *σ*^54^-RNAP holoenzyme forms an inactive, stable closed complex in bacteria^52^, whereas negative elongation factors cause RNAP to stall within 20–60 bases downstream of the transcription start site in many metazoan genes^53^. In all cases, inhibited RNAPs are ready-to-fire when activating signals arrive from relevant signal-transduction cascades thus avoiding promoter search, binding, melting, and activation.

## Methods

### Glass coverslips preparation for single-molecule experiments

Borosilicate glass coverslips (1.5 MenzelGläzer, Germany) were sonicated for 30 min in a 2% (V/V) solution of Hellmanex III (Helma Analytics, Germany)/deionized water. After being thoroughly rinsed with deionized water, the coverslips were dried, disposed into a plasma cleaner (Harrick Plasma, NY, USA), and exposed to a nitrogen plasma for 30 min. The coverslips are subsequently immerged into a 1% (V/V) solution of Vectabond (product code SP-1800, Vector Labs, CA, USA)/acetone for 10 min. The coverslips were then rinsed in deionized water and dried with a stream of nitrogen gas. After disposing a silicone gasket (103280, Grace Bio-Labs, OR, USA) on each coverslip, each well was filled with 20 µl of a 100 mg/ml solution of methoxy-PEG (5 kDa)-SVA/ biotin-PEG (5 kDa)-SC (2.5% (w/w) (Laysan Bio, AL, USA) in 50 mM MOPS-KOH buffer, pH 7.5 The wells were incubated for ~ 1.5 h and thoroughly rinsed with a 1 × phosphate-buffered saline (PBS; Sigma Aldrich, UK) solution. The coverslips were stored at 4 °C up to 2 weeks before use.

### Protein immobilization protocol

The pegylated coverslips were incubated for ~ 10 min with a solution 0.5 mg/ml of Neutravidin (31050, Sigma Aldrich) in 0.5 × PBS and subsequently rinsed with 1 × PBS. Preceding observation on the microscope, the coverslips were incubated for ten min with a 3% (V/V) solution of Penta•His biotin conjugate antibody (34440, QIAGEN, UK) in reaction buffer (40 mM HEPES buffer pH 7.3 (ThermoFisher Scientific, UK), 100 mM potassium glutamate, 10 mM MgCl_2_, 1 mM dithiothreitol (DTT), 1 mM cysteamine hydrochloride, 5% glycerol (V/V), 0.5 g/l bovine serum albumin) and subsequently rinsed with reaction buffer. After adjusting the coverslips on the microscope, 100 pM of His-tagged protein–DNA complex, e.g., RPo, was incubated in the observed well until the desired density of molecules on the coverslips surface was reached, followed by one-step rinsing with reaction buffer.

### Core RNAP and *σ*^70^ preparation

The expression and purification of the core bacterial RNAP have previously been described in ref. ^54^. The expression and purification of the WT *σ*^70^ have been previously described in ref. ^21^.

### Holo-RNAP and RP_O_ assembly

Core RNAP (0.5 µM) was mixed with 0.6 µM of *σ*^70^ in 20 mM Tris-HCl pH 7.9, 150 mM NaCl, 0.1 mM EDTA, 50% (V/V) Glycerol, and 0.1 mM DTT, and incubated at 30 °C for 30 min. The resulting holo-complex was stored at – 20 °C.

Holo-complex (5 nM) was mixed with 2.5 nM of DNA promoter in reaction buffer and incubated for 10 min at 37 °C to form the RPo complex.

### DNA constructs preparation

The DNA constructs preparation is described in detail in the Supplementary Methods.

### Microscope and single-molecule experiments

The single-molecule TIRF microscope for FRET experiments has been previously described in ref. ^55^. Shortly, the 532 nm and the 642 nm wavelength laser beams (donor laser excitation and acceptor laser excitation, respectively) were focused in the back focal plane of an oil immersion objective (Olympus, N.A. 1.4) and illuminate alternatively the field of view, i.e., ALEX mode^56^. The TIRF-reflected beams were directed toward a position sensor to control the objective focal plane distance to the sample at a fixed position (MS-2000 stage, ASI, OR, USA). The photons resulting from the de-excitation of the dyes molecules, i.e., fluorescence, were separated from the excitation laser beams with a dichroic mirror and spectrally splitted in two channels, e.g., donor and acceptor that are imaged on the same electron-modifying charge-coupled device camera (iXon, Andor, Irlande). For 100 ms ALEX illumination, i.e., 200 ms frame time acquisition, the laser power measured preceding the dichroic mirror is ~ 0.4 mW for donor excitation laser and ~ 0.09 mW for the acceptor excitation laser. For 40 ms ALEX illumination (only used to acquire the data with the ΔP promoter and ATP starting substrate experimental condition), i.e., 80 ms frame time, the laser power measured preceding the dichroic mirror is ~ 1 mW for donor excitation laser and ~ 0.25 mW for the acceptor excitation laser.

The imaging buffer contained the reaction buffer completed with 1 mM Trolox, 1 mM COT, 1% (w/V) glucose, 0.4 µg/ml of catalase, and 1 mg/ml of glucose oxidase (Sigma Aldrich). The catalase and the glucose oxidase were pre-mixed together in a solution of 50 mM KCl and 50 mM Tris-OAc buffer pH 7.3 at 100 × concentration^57,58^.

The data were acquired after immobilization of the RPo complex to the surface. After ~ 200 frames (~ 20 s), the imaging buffer is spiked with a 12.5 × NTP solution and the reaction is observed for the remaining ~ 5800 frames (total time: 10 min).

For the post-RNA synthesis rinsing experiments, the RP_O_ was incubated with NTPs in the reaction buffer for 10 s before the reaction buffer was exchanged twice and finally replaced with imaging buffer, followed by the start of the acquisition. The buffer exchange procedure takes ~40 sec to be completed before the start of the acquisition.

All single-molecule FRET experiments were performed at 22 °C.

### Single-molecule data analysis

FRET pair localization and detection: The movies recorded on the camera were offline analyzed using the home-built Matlab routine Twotone-ALEX^59^ to extract the intensities of co-localized donor and acceptor, i.e., FRET-pair. The following parameters from Twotone-ALEX were used to select only the FRET pairs formed by a single ATTO647N acceptor dye and a single Cy3b donor dye: channel filter as DexDem&&AexAem&&DexAem (colocalization of the donor dye signal upon donor laser excitation, the acceptor dye signal upon acceptor laser excitation, and the acceptor dye signal upon donor laser excitation), a width limit between the donor and the acceptor between 1 and 2 pixels, a nearest-neighbor limit of 6 pixels, and a maximal ellipticity of 0.6 (ellipticity is defined as the ratio of the minor and the major axis of the ellipse). The traces extracted from the Twotone-ALEX analysis were then sorted to remove all the traces that displayed extensive blinking or multisteps photobleaching, i.e., that contain more than one donor or acceptor dye in the same diffraction limited intensity spot.

### Calibration of the FRET sensor

We calibrated the FRET sensor by measuring *E*_FRET_ for initial transcribing complexes in the presence of different subsets of nucleotides that allowed maximal transcript lengths of 6, 7, or 11 nucleotides, and compared their FRET profiles with RP_O_. These experiments allowed unambiguous assignment of the RP_O_ and ITC11 FRET levels (*E*_FRET_) as ~ 0.5 and ~ 0.76, respectively (Fig. 1c; see also Supplementary Fig. 1). For clarity, we define these FRET states as “US” and “FS”, respectively. Although it is commonly accepted that RNAP escapes *lac* promoters after synthesizing an 11-mer^9,60^, the high FRET signal after forming a 11-nt long transcript (Fig. 1c) is consistent with extended scrunching of the downstream DNA in an ITC (and not an elongation complex). On the other hand, complexes with maximal transcript lengths of 6 and 7 nucleotides yielded *E*_FRET_ 0.38 and 0.37, respectively. We observed here a decrease in *E*_F__RET_, i.e., an increase in the inter-dye distance as a consequence of further transcript extension as the downstream dye at some positions moves farther along from the donor due to the rotation of the downstream dsDNA^27,61^. The assignment of this *E*_FRET_ level to an ITC6 pause state, or “PS,” was based on our previous observations showing that ITC6 (but not ITC7) accumulates in significant amounts in conditions that allow continuous transcription past the 6–7-mer RNA and that ITCs do not pause again until reaching the position + 12 of the DNA template used here^27^.

### FRET efficiency and hidden Markov modeling

The FRET efficiency dynamics for each FRET-pair was calculated with the standard formula $E = \frac{{I_{\mathrm{{DA}}}}}{{I_{\mathrm{{DA}}} + I_{\mathrm{{DD}}}}}$, with *I*_DA_ and *I*_DD_ being respectively the intensity of the acceptor and of the donor upon donor excitation^62^. The traces were analyzed through a modified version of the hidden Markov model ebFRET software from ref. ^63^ (the modified code is available from the corresponding authors on reasonable request), where only steps longer than two frames and separated from the subsequent step by more than twice the Allan deviation estimated at five frames were conserved^64^ to be assembled into dwell time. The first dwell time, i.e., preceding NTP addition, and the last dwell time, i.e., preceding photobleaching or transition to FS FRET state, of each trace were removed from the dwell time distribution.

### Characterization of the dwell time distributions

A detailed analysis of the dwell time distributions is provided in ref. ^65^. Shortly, the distribution of $\tau$ are described by a probability distribution function with *m* exponentials:

1 $$p_t\left( \tau \right) = \mathop {\sum }\limits_{n = 1}^m p_n \cdot k_n \cdot e^{ - k_n \cdot \tau },$$

where *k*_*n*_ and *p*_*n*_ are the characteristic rate of the *m*^th^ exponential and its probability, respectively. The minimum number of exponential to fit the distributions was determined for each distribution by using the Bayes Schwarz Information Criterion^66^. We calculate the maximum likelihood estimate of the parameters (MLE)^67^ by maximizing:

2 $$L = \mathop {\sum }\limits_{i = 1}^N {\mathrm{ln}}(p_t\left( {\tau _i} \right))$$

over the parameter set. Here, the $\tau _i$ are the experimentally measured dwell times and *N* is the number of collected dwell times $\tau _i$. The error bars for each fitting parameters are one standard deviation extracted from 1000 bootstrap procedures^68^. The ebFRET software^63^ was also used to extract the peak positions of each FRET level, subsequently fitted with a Gaussian function, with the peak center and the SD as free parameters (Supplementary Figs. 1d and 3a-d).

### Data availability

The data sets generated and analyzed during the current study are available from the corresponding author on reasonable request.

## Electronic supplementary material

Supplementary Information
