# Supplementary material for: Pausing controls branching between productive and non-productive pathways during initial transcription in bacteria
Source: Nat Commun. 2018 Apr 16;9:1478. doi: 10.1038/s41467-018-03902-9 (PMC5902446; doi:10.1038/s41467-018-03902-9)
Supplement: Supplementary file 1 — Supplementary Information [file 41467_2018_3902_MOESM1_ESM.pdf]

Supplementary Information for:

Pausing controls branching between productive and non-productive pathways during initial transcription in bacteria

Dulin et al.

Supplementary Methods

DNA constructs fabrication

The promoter DNA fragments (**Supplementary Figure 1a**) were prepared in a modular fashion, *i.e.* modular fragments of oligonucleotides (oligo 1, 2 and 3) were mixed with a complementary splint DNA to form a ligation-competent complex (LCC). Template and non-template strands were created separately. Oligo 2 was purchased from IBA Life Science (Göttingen, Germany), and the splint, oligo 1 and 3 were purchased from Sigma Aldrich (Poole, UK) and stored in TE buffer at -20°C (100 µM).

DNA preparation: 1µl of a 10x T4 DNA Ligase Reaction Buffer and 1µl of a T4 Polynucleotide Kinase (final conc. 1,000 units/ml) were mixed together with 1µl of each oligonucleotide that did not provide the 5-end of the full-length product. The mixture was filled up with water to a total volume of 10µl and incubated at 37°C for 90min. Afterwards, the sample was heat-inactivated. 1µl of the splint and the non-phosphorylated oligonucleotide were added together with 12 µl of a 2x T7 DNA Ligase Buffer to anneal the LCC in a thermocycler. Finally, ligation was performed by adding 1µl T7 DNA ligase (final conc. 120,000 units/ml) and incubate at 15°C over night. Enzymes and buffers were purchased from NEB.

Purification: to separate the ligated DNA strand from the splint and incomplete ligation products, we used a denaturing 10% polyacrylamide gel (14.5cm x 16.5cm x 1.5mm). While prerunning the gel in 1xTBE, the sample was mixed with formamide (85%), heated up to 95°C for 5 min, and immediately put on ice. The gel was run at 450V for 90min. Bands of the full-length product were excised and the strands eluted by crush-and-soak overnight in 1x TBE buffer and ethanol precipitated.

Template and non-template DNA strands assembly: the DNA promoters were created by annealing equimolar amounts of template and non-template strand oligonucleotides in buffer TE-50 (50 mM Tris pH 7.4, 1 mM EDTA, 50 mM NaCl) at 400 nM.

The oligonucleotides are represented in the 5' to 3' direction.

Oligonucleotide sequences for the non-template strands (name of the oligo, promoter, position within the promoter, length)

Splint WT and ds (-79,+25), 104 nt
GAAATTGTTATCCGCTCTCACAATTCCACACATTATACGAGCCGAAGCATAAAGTGTCAAGCCTGGGGTGC
CTAAAATGTGATCTAGATCACATTTATTGCGTT

Splint -7T/A (-79,+25), 104 nt
GAAATTGTTATCCGCTCTCACAATTCCACACTTTATACGAGCCGAAGCATAAAGTGTCAAGCCTGGGGTGC
CTAAAATGTGATCTAGATCACATTTATTGCGTT

Splint WT ITC6 (-79,+25), 104 nt
GAAATTGTTATCCGCTCCTACAATTCCACACATTATACGAGCCGAAGCATAAAGTGTCAAGCCTGGGGTGC
CTAAAATGTGATCTAGATCACATTTATTGCGTT

Splint ΔP (-79,+25), 104 nt
GAAATTGTTATCCGCTCTACCAATTCCACACATTATACGAGCCGAAGCATAAAGTGTCAAGCCTGGGGTGC
CTAAAATGTGATCTAGATCACATTTATTGCGTT

Oligo 3 (-89,-30), 60 nt
CAGTGAGCGCAACGCAATAAATGTGATCTAGATCACATTTTAGGCACCCCAGGCTTGACA

Oligo 2 (-29,-10), 20 nt
CTTTATGCTTCGGCXCGTAT with X=T-Cy3B

Oligo 1 WT and ds (-9,+25), 34 nt
AATGTGTGGAATTGTGAGAGCGGATAACAATTTC

Oligo 1 -7T/A (-9,+25), 34 nt
AAAGTGTGGAATTGTGAGAGCGGATAACAATTTC

Oligo 1 WT ITC6 (-9,+25), 34 nt
AATGTGTGGAATTGTAGGAGCGGATAACAATTTC

Oligo 1 ΔP (-9,+25), 34 nt
AATGTGTGGAATTGGTAGAGCGGATAACAATTTC

Oligonucleotides for WT template strand

Splint (-79,+25), 104 nt
AACGCAATAAATGTGATCTAGATCACATTTTAGGCACCCCAGGCTTGACACTTTATGCTTCGGCTCGTAGC
CGTGTTGGAATTGTGAGAGCGGATAACAATTTC

Oligo 1 (+8,+25), 18 nt
GAAATTGTTAXCCGCTCT with X=T-ATTO647N

Oligo 2 (-29,+7), 36 nt
CACAATTCCAACACGGCTACGAGCCGAAGCATAAAG

Oligo 3 (-89,-30), 60 nt
TGTCAAGCCTGGGGTGCCTAAAATGTGATCTAGATCACATTTATTGCGTTGCGCTCACTG

Oligonucleotides for WT ITC6 template strand

Splint (-79,+25), 104 nt
AACGCAATAAATGTGATCTAGATCACATTTTAGGCACCCCAGGCTTGACACTTTATGCTTCGGCTCGTAGC
CGTGTTGGAATTGTAGGAGCGGATAACAATTTC

Oligo 1(+8,+25), 18 nt
GAAATTGTTAXCCGCTCC with X=T-ATTO647N

Oligo 2 (-29,+7), 36 nt
TACAATTCCAACACGGCTACGAGCCGAAGCATAAAG

Oligo 3 (-89,-30), 60 nt
TGTCAAGCCTGGGGTGCCTAAAATGTGATCTAGATCACATTTATTGCGTTGCGCTCACTG

Oligonucleotides for ΔP template strand

Splint (-79,+25), 104 nt
AACGCAATAAATGTGATCTAGATCACATTTTAGGCACCCCAGGCTTGACACTTTATGCTTCGGCTCGTAGC
CGTGTTGGAATTGGTAGAGCGGATAACAATTTC

Oligo 1 (+8,+25), 18 nt
GAAATTGTTAXCCGCTCT with X=T-ATTO647N

Oligo 2 (-29,+7), 36 nt
ACCAATTCCAACACGGCTACGAGCCGAAGCATAAAG

Oligo 3 (-89,-30), 60 nt
TGTCAAGCCTGGGGTGCCTAAAATGTGATCTAGATCACATTTATTGCGTTGCGCTCACTG

Oligonucleotides for ds template strand

Splint (-79,+25), 104 nt
AACGCAATAAATGTGATCTAGATCACATTTTAGGCACCCCAGGCTTGACACTTTATGCTTCGGCTCGTATA
ATGTGTGGAATTGTGAGAGCGGATAACAATTTC

Oligo 1 (+8,+25), 18 nt
GAAATTGTTAXCCGCTCT with X=T-ATTO647N

Oligo 2 (-29,+7), 36 nt
CACAATTCCACACATTATACGAGCCGAAGCATAAAG

Oligo 3 (-89,-30), 60 nt
TGTCAAGCCTGGGGTGCCTAAAATGTGATCTAGATCACATTTATTGCGTTGCGCTCACTG

Oligos for bead-attached bulk transcription experiments:

Oligo non-template DNA (-39, +25), 64 nt
XGGCTTGACACTTTATGCTTCGGCTCGTATAATGTGTGGAATTGTGAGAGCGGATAACAATTTC
with X=A-BIOTIN

Oligo template DNA (-39, +25), 64 nt
GAAATTGTTATCCGCTCTCACAATTCCAACACGGCTACGAGCCGAAGCATAAAGTGTCAAGCCT

Protocols for in vitro bulk transcription experiments

Enzymes & transcription templates. *In vitro* transcription reactions were performed with RNAP holoenzyme purchased from Epicentre or New England Biolabs or obtained as described previously (Pupov et al., 2014). For FRET measurements, σ^{70} was purified as described [1] and incubated at 2-fold excess with the core enzyme for 30 min at 30 ºC to form a holoenzyme, which was stored in RNAP storage buffer (20 mM Tris-HCl pH 7.9, 150 mM NaCl, 0.1 mM EDTA, 0.1 mM DTT, 50% v/v Glycerol) at -20 ºC until needed. Sequences of transcription promoter-templates and oligos are provided in **Supplementary Figure 1a**. Analysis of mutant RNAP activities (**Figure 3b**) was performed with the full-length WT consensus *lac* promoter fragment spanning positions from -89 to +25. The RNA retention and extension experiments were performed with a shorter version of the WT promoter, from -39 to +25, that was created by annealing equimolar amounts of template and non-template strand oligonucleotides[2] in buffer TE-50 (50 mM Tris pH 7.4, 1 mM EDTA, 50 mM NaCl) at 100-400 nM as required.

***In vitro* transcription reactions.**
For control experiments on bead-bound transcription complexes (**Figure 5k**, **Supplementary Figure 4ab**), 2.5 µl streptavidin-coated magnetic beads (DynaBeads, Life Technologies) were equilibrated in 1x KG7 buffer (40 mM HEPES buffer pH 7.3 (ThermoFisher Scientific, UK), 100 mM potassium glutamate, 10 mM $MgCl_2$, 1 mM dithiothreitol (DTT), 1 mM cysteamine hydrochloride, 5% glycerol (V/V), 0.5 g/l BSA), pre-formed RP_O was bound at 37ºC for 15 min with gentle agitation, and beads were washed 3x with 1x KG7. The experiment from **Supplementary Figure 4c** was performed with complexes adsorbed on Ni-NTA-agarose resin (Qiagen). Transcription was initiated by the addition of the reaction mix shown below and agitated gently. The reaction mix contained: 4 units of RNAsin (Promega, USA), 0.1 mg/mL heparin, 500 µM ApA, 0.6 µCi/µL [α-^{32}P]-UTP (Perkin Elmer), and 80 µM of either UTP + GTP to reach 7 nt or UTP, GTP and ATP for 11-mer synthesis in 1x KG7 buffer (all concentrations are final). For released vs. retained experiments, reactions were halted after a 10-sec incubation (**Supplementary Figure 4ab**) or 1 min incubation at 37 °C (Figure VIII-short – will be **Supplementary Figure 4c**) by pulling down the transcription complexes, then one time rinsed with KG7 1x buffer and disposed in KG7 1x buffer for the indicated duration at 22 °C. Subsequently, the transcription complexes were pulled-down, to run on a PAGE gel the released transcripts collected from the supernatant (Released Fraction, **Supplementary Figure 4**) and the transcripts retained in the transcription complexes (Retained Fraction, **Supplementary Figure 4**).

Transcription reactions with mutant RNAPs (**Figure 3b**) were performed in 1x KG7 buffer at 37 ºC. Promoter complexes were formed for 5 minutes with 200 nM core RNAP, 500 nM σ^{70} subunit or its mutant variants and 500 nM promoter DNA fragments, followed by NTP addition (500 µM ATP and 80 µM each GTP, UTP; or 500 µM dinucleotide primer ApA and 80 µM ATP, GTP, UTP); transcription was performed for 1 minute at 37ºC.

In all experiments, FLB-stop buffer (86% formamide, 20 mM EDTA, 1x TBE, amaranth dye) was added to the beads directly to quench the reactions and the mixture was heated to 95ºC for 4 min, placed on ice, and the supernatant was loaded directly onto a high-resolution sequencing gel (30% 19:1 acrylamide/bis-acrylamide gel, 6 M urea, 1x TBE) and electrophoresed in 1x TBE at 32 V/cm for 30 min until samples entered the gel, then at 75 V/cm until the amaranth dye had run ~85% of the gel length. Typical running time for a 40-cm gel was 3000 V for 3.5 hours. The gel was cut to remove unincorporated radioactive NTPs (typically 1 cm above the amaranth dye band).

Supplementary Note

Evaluation of the population that does not pause at ITC6
Interestingly, we observed that 3–20% of the ITCs did not display a pause in the PS state (plain bars, **Supplementary Figure 2b**), but rather a direct transition from US to FS. This indicates two possible origins for the apparent absence of pausing: the presence of a non-pausing population of ITCs, and inadequate temporal resolution to capture the fastest US→PS→FS transitions. We thus calculated the fraction of the pausing ITCs that we cannot technically detect (by integrating the pause-exit probability distribution from 0 to our detection limit, which is described in **FRET efficiency characterization and Hidden Markov Modeling of the single-molecule FRET traces** paragraph, **Materials and Methods** section), and subtracted this fraction from the total non-pausing ITC population (plain bars, **Supplementary Figure 2b**). The corrected populations (dashed bars, **Supplementary Figure 2b**) showed that, for the WT promoter (for ATP and ApA starting substrate), the non-pausing events arise mainly due to limited resolution; in contrast, for the ΔP promoter, the main reason is actually the presence of non-pausing RPs (**Supplementary Figure 2b**). The $T^{+6}G^{+7}$ (ntDNA) sequence therefore enforces pausing at ITC6 for ~100% of ITCs, stabilizing the pre-translocated state arising from the clash between $\sigma_{3.2}$ and the 5'-RNA end [2,3].

Supplementary Discussion

Backtracking in transcription initiation
We have identified here cycles of unscrunching/scrunching in transcription complexes that have failed in escaping the ITC6 pause on the first attempt. Similar backtracked/unscrunched initially transcribing complexes were recently identified in a magnetic tweezers assay [4]. Both that study and our current work described the US state kinetics with a double-exponential distribution, with the longest-lived one being the backtracked complexes. Lerner et al. [4] reported backtracked complex lifetimes two orders of magnitude longer than we find in our study. However, the difference can be easily explained as the magnetic tweezers assay of Lerner et al. is based on the transcription-dependent changes in the supercoil density of a positively supercoiled DNA. Essentially, RNAP activity, e.g. open complex formation and RNA synthesis, generates plectonemes ("DNA loops") [4-7]. However, the energetic cost of plectoneme formation [8-11] biases the transcribing complex towards less extensive scrunching, increasing the lifetime of the backtracked/unscrunched state. Our FRET assay, in contrast, employed non-supercoiled DNA and reported a shorter lifetime of the backtracked/unscrunched state. In addition, the symmetry in the dynamics of the US and the PS states, together with its independence on catalytic activity or the nature of the interactions between the RNAP and the promoter, suggest that the transition between the states described by two exponentials, i.e. described by exit rates k_1 and k_2, (**Figure 4 and Supplementary Figure 2hm**) originates from a conformational change within the holoenzyme that precludes catalysis but does not preclude the dynamics of the hybrid, i.e. scrunching/unscrunching cycle.
We note that the presence of the GreA and GreB factors *in vivo* likely shortens the lifetime of the backtracked US state by cleaving the protruding 3'-RNA end of backtracked complexes [12], as observed by Lerner et al. in *in vitro* single-molecule experiments [4]. Unfortunately, we could not

evaluate the effects of Gre-factors with our single-molecule FRET assay because their binding to RP caused extensive flickering of the Cy3B dye.

Moribund complexes and the cyclically unscrunching/scrunching states

Henderson et al. reported that the moribund complexes on their promoters and under their conditions were not able to elongate their transcripts [13]. In contrast, our complexes are able to extend the retained RNA (**Figure 5k**), and could enter the cyclic unscrunching/scrunching state multiple times before elongating the transcript, as a function of the NTP concentration (**Figure 1d, Figure 2c**). This conclusion is further supported by the biphasic activity also observed with purified moribund complexes [14,15], demonstrating that moribund complexes have entered a catalytically incompetent state, which is eventually exited towards productive synthesis. Supporting our conclusion on the backtracked nature of the US state, Shimamoto and co-workers have also observed that the moribund complexes were reactivated by GreA [14]. They also showed that part of the moribund complexes are converted into dead-end complexes on λP_R promoter, i.e. that could not show any catalytic activity anymore, while trapping a short transcript [15]. Interestingly, their gels showed that a large amount of 9-mer transcript was produced, despite the presence of all NTPs, which correlated with the presence of a (YG) sequence on the non-template promoter DNA at the +9 position. We showed here that such a (YG) sequence increased the probability to enter the cyclic unscrunching/scrunching pathway (**Figure 2c**). We suggest that the dead-end complexes observed on the λP_R promoter are the consequence of multiple successive entries into the cyclic unscrunching/scrunching pathway, and therefore appeared inactivated. Similarly, it is likely that the complexes observed by Henderson et al. [13] have not yet recovered from the cyclic unscrunching/scrunching state. Nevertheless, our data clearly demonstrate that the unscrunching of the promoter DNA is not linked with obligatory abortive transcription and the release of RNA. Instead, off-pathway ITCs can sample different scrunched conformations and eventually resume productive RNA synthesis. Such conformational changes have also been proposed for promoter-proximal paused complexes [16].

A model for initial transcription

In the RP_O complex, $\sigma_{3.2}$ interacts with and stabilises the melted template DNA strand in the DNA binding cleft of RNAP. Upon NTP addition, RNAP engages RNA synthesis producing rapidly either abortive 2–5-mer RNAs or ITC6. In ITC6 stage, $\sigma_{3.2}$ becomes a barrier to the 5'-end of RNA stabilising the pre-translocated ITC6 – the key intermediate of our model that can branch to two competing reaction pathways. The fraction of ITC6 that remain on the productive pathway eventually overcome the barrier for translocation, possibly via rate-limiting retraction of the $\sigma_{3.2}$ tip towards the RNA exit channel, resuming RNA synthesis. The pre-translocated ITC6 may also embark on an alternative off-pathway by isomerisation into the initial paused conformation ($\mathrm{ITC6}_{\mathrm{pre}}^{\mathrm{inactive}}$ in **Figure 6**). The paused state is further stabilised by the cyclic unscrunching/scrunching (or backtracking/forward-translocation) events depicted as "Futile cycling" in **Figure 6**, which does not involve RNA synthesis or RNA release. The fact that the dwell-time distributions of both the PS and US states were double-exponential suggest that they can be further stabilized into long-lived PS ($\mathrm{ITC6}_{\mathrm{pre}}^{\mathrm{inactive,stable}}$) and US ($\mathrm{ITC6}_{\mathrm{backtr}}^{\mathrm{inactive,stable}}$) states, respectively. These states can retain the transcript for >45 min (**Supplementary Figure 4**) and ultimately return to the productive pathway and escape the ITC6 pause. The model we present here contains three significant molecular mechanisms, i.e. the initial barrier imposed by $\sigma_{3.2}$ to the transcript elongation (1 in **Figure 6**), the subsequent loss of catalytic conformation (2 in **Figure 6**) and the RNA-dependent reversible backtracking (3 in **Figure 6**), which potentiate, initiate and amplify the pause encountered by the initially transcribing bacterial RNAP, respectively.

Supplementary Table 1: Statistics, exit rates and probabilities for all the experiments performed in single-molecule FRET.

Experimental conditions (promoter, core RNAP, σ, starting substrate, NTPs, rinsed)	Figures	Number of traces; with FS FRET levels	IPE number of points; k_{IPE} (s^{-1})	US number of points; k_1 (s^{-1}); k_2 (s^{-1}); $P(k_1)$	PS number of points; k_1 (s^{-1}); k_2 (s^{-1}); $P(k_1)$	FS number of points; k_1 (s^{-1}); k_2 (s^{-1}); $P(k_1)$
WT, WT, WT, ApA, all NTPs 1μM, no	2, 4, S2	151; 8	8; (*)	311; 0.11; 0.013;0.5	382; 0.11; 0.012;0.64	(**)
WT, WT, WT, ApA, all NTPs 10μM, no	2, 4, S2	176; 44	38; 0.046	223; 0.09; 0.014;0.57	278; 0.27; 0.013;0.21	(**)
WT, WT, WT, ApA, all NTPs 30μM, no	2, 4, S2	317; 135	123; 0.041	238; 0.17; 0.017;0.65	283; 0.13; 0.015;0.25	(**)
WT, WT, WT, ApA, all NTPs 50μM, no	2, 4, S2	310; 162	148; 0.037	184; 0.20; 0.032;0.63	218; 0.24; 0.017;0.20	(**)
WT, WT, WT, ApA, all NTPs 80μM, no	2, 4, S2	599; 363	308; 0.049	313; 0.22; 0.029;0.46	262; 0.22; 0.019;0.07	(**)
WT, WT, WT, ApA, ITC11 80μM, no	3	238; 167	149; 0.052	(**)	(**)	(**)
WT, WT, WT, ApA, all NTPs 500μM, no	2, 4, S2	330; 266	233; 0.046	(*)	(*)	(**)
WT, WT, WT, ATP, ITC11 5μM, no	2, 4, S2	94; 28	18; 0.079	151; 0.14; 0.018;0.49	176; 0.10; 0.011;0.31	(**)
WT, WT, WT, ATP, ITC11 10μM, no	2, 4, S2	88; 33	29; 0.094	103; 0.16; 0.014;0.46	133; 0.12; 0.011;0.57	(**)
WT, WT, WT, ATP, ITC11 30μM, no	2, 4, S2	230; 143	121; 0.10	151; 0.08; 0.012;0.68	174; 0.10; 0.018;0.47	(**)
WT, WT, WT, ATP, ITC11 50μM, no	2, 4, S2	211; 122	94; 0.115	160; 0.10; 0.014;0.55	177; 0.07; 0.014;0.58	(**)
WT, WT, WT, ATP, ITC11 80μM, no	2, 4, S2	246; 150	128; 0.099	139; 0.12; 0.014;0.56	159; 0.08; 0.015;0.65	(**)
WT, WT, WT, ATP, ITC11 80μM, no	*5, S3*	*79; (**)*	*(**)*	*681; 0.93; 0.057;0.87*	*115; 0.59; 0.045;0.36*	*596; 0.32; 0.059; 0.81*
WT, WT, WT, ATP, ITC11 500μM, no	2, 4, S2	191; 141	120; 0.155	(*)	(*)	(**)
ΔP, WT, WT, ATP, ITC11 5μM, no	2, 4, S2	88; 55	42; 0.18	(*)	(*)	(**)
ΔP, WT, WT, ATP, ITC11 10μM, no	2, 4, S2	94; 60	53; 0.19	(*)	(*)	(**)
ΔP, WT, WT, ATP, ITC11 30μM, no	2, 4, S2	85; 66	52; 0.29	(*)	(*)	(**)
ΔP, WT, WT, ATP, ITC11 50μM, no	2, 4, S2	245; 190	147; 0.21	(*)	(*)	(**)
ΔP, WT, WT, ATP, ITC11 80μM, no	2, 4, S2	152; 123	94; 0.32	(*)	(*)	(**)
ΔP, WT, WT, ATP, ITC11 500μM, no	2, 4, S2	97; 76	60; 0.33	(*)	(*)	(**)
ΔP, WT, WT, ApA, all NTPs 1μM, no	2, 4, S2	96; 3	3; (*)	238; 0.15; 0.018;0.53	289;0.096; 0.018;0.53	(**)
ΔP, WT, WT, ApA, all NTPs 5μM, no	2, 4, S2	148; 49	39; 0.057	285; 0.22; 0.020;0.47	327;0.28; 0.024;0.27	(**)
ΔP, WT, WT, ApA, all NTPs 10μM, no	2, 4, S2	162; 71	66; 0.064	189; 0.12; 0.013;0.76	216; 0.098; 0.020;0.46	(**)
ΔP, WT, WT, ApA, all NTPs 30μM, no	2, 4, S2	328; 220	186; 0.051	210; 0.14; 0.026;0.59	249; 0.20; 0.030;0.36	(**)
ΔP, WT, WT, ApA, all NTPs 50μM, no	2, 4, S2	311; 210	172; 0.06	213; 0.19; 0.025;0.68	237; 0.11; 0.025;0.56	(**)
ΔP, WT, WT, ApA, all NTPs 80μM, no	2, 4, S2	302; 209	164; 0.085	123; 0.13; 0.015;0.7	130; 0.10; 0.025;0.62	(**)
ΔP, WT, WT, ApA, all NTPs 500μM, no	2, 4, S2	326; 258	226; 0.075	87;0.19;0.04;0.89	109;0.08;0.026;0.56	(**)
WT, WT, F522A, ApA, ITC11 80μM, no	3, S2	207; 78	67; 0.047	107; 0.12; 0.018;0.75	134; 0.10; 0.022;0.45	(**)
WT, WT, F522A, ATP, ITC11 80μM, no	S2	289; 119	49; 0.15	133; 0.21; 0.024;0.63	153; 0.34; 0.024;0.67	(**)
WT, D446A, WT, ApA, ITC11 30μM, no	S2	53; 6	6; (*)	(*)	(*)	(**)
WT, D446A, WT, ATP, ITC11 30μM, no	S2	37; 12	12; (*)	121; 0.15; 0.031;0.51	145; 0.19; 0.017;0.70	(**)
WT, D446A, WT, ApA, ITC11 500μM, no	3, S2	127; 27	15; 0.014	121; 0.15; 0.031;0.51	145; 0.19; 0.017;0.70	(**)
WT, D446A, WT, ATP, ITC11 500μM, no	S2	137; 47	29; 0.055	158; 0.13; 0.018;0.67	179; 0.13; 0.016;0.65	(**)
-7T/A, WT, WT, ApA, ITC11 80μM, no	S2	256; 139	131; 0.036	169; 0.13; 0.016;0.82	196; 0.29; 0.018;0.56	(**)
dsWT, WT, WT, ApA, ITC11 80μM, no	S2	148; 21	21; 0.037	313; 0.22; 0.029;0.46	262; 0.22; 0.019;0.07	(**)
dsWT, WT, WT, ATP, ITC11 80μM, no	S2	132; 77	64; 0.104	(*)	(*)	(**)
WT, WT, WT, ApA, ITC7 80μM, no	S2	221; 0	(***)	544; 0.19; 0.024;0.53	633; 0.22; 0.022;0.15	(**)
WT, WT, WT, ApA, ITC7 80μM, yes	5, S3	70; 0	(***)	225; 0.11; 0.023;0.60	209; 0.14; 0.019;0.51	(**)
WT, WT, WT, ApA, ITC11 80μM, yes	5, S3	99; 48	(***)	244; 0.10; 0.016;0.65	208; 0.15; 0.019;0.42	(***)
WT, WT, WT, ApA, ITC11 80μM, yes	5, S3	99; 48	(***)	396; 1.0; 0.06;0.78	84; 0.12; 0.028;0.59	313; 0.36; 0.075;0.64
WT, WT, WT, ATP, ITC11 80μM, yes	5, S3	129; 79	(***)	383; 0.26; 0.031;0.52	402; 0.26; 0.024;0.78	(***)
WT, WT, WT, ATP, ITC11 80μM, yes	5, S3	129; 79	(***)	763; 0.95; 0.081;0.73	236; 0.39; 0.049;0.67	617; 0.68; 0.091;0.71

(*) Not determined because the statistics was insufficient

(**) Not Relevant.

(***) Not existing in these experimental conditions.

In italic: same dataset as the line above, kinetics after reaching the FS FRET level the first time.

a

T(-15)-Cy3B

WT (premelted)

5′ CAGTGAGCGCAACGCAATAAATGTGATCTAGATCACATTTTAGGCACCCCAGGCTTGACACTTTATGCTTCGGCTCGTATAATGTGTGGAATTGTGAGAGCGGATAACAATTTC 3′
3′ GTCACTCGCGTTGCGTTATTTACACTAGATCTAGTGTAAAATCCGTGGGGTCCGAACTGTGAAATACGAAGCCGAGCATCGGCACAACCTTAACACTCTCGCCTATTGTTAAAG 5′

-35 element -10 element

T(+15)-ATTO647N

WT (premelted) for ITC6

5′ CAGTGAGCGCAACGCAATAAATGTGATCTAGATCACATTTTAGGCACCCCAGGCTTGACACTTTATGCTTCGGCTCGTATAATGTGTGGAATTGTAGGAGCGGATAACAATTTC 3′
3′ GTCACTCGCGTTGCGTTATTTACACTAGATCTAGTGTAAAATCCGTGGGGTCCGAACTGTGAAATACGAAGCCGAGCATCGGCACAACCTTAACATCCTCGCCTATTGTTAAAG 5′

ΔP DNA promoter (premelted)

5′ CAGTGAGCGCAACGCAATAAATGTGATCTAGATCACATTTTAGGCACCCCAGGCTTGACACTTTATGCTTCGGCTCGTATAATGTGTGGAATTGGTAGAGCGGATAACAATTTC 3′
3′ GTCACTCGCGTTGCGTTATTTACACTAGATCTAGTGTAAAATCCGTGGGGTCCGAACTGTGAAATACGAAGCCGAGCATCGGCACAACCTTAACCATCTCGCCTATTGTTAAAG 5′

ds (double-stranded)

5′ CAGTGAGCGCAACGCAATAAATGTGATCTAGATCACATTTTAGGCACCCCAGGCTTGACACTTTATGCTTCGGCTCGTATAATGTGTGGAATTGTGAGAGCGGATAACAATTTC 3′
3′ GTCACTCGCGTTGCGTTATTTACACTAGATCTAGTGTAAAATCCGTGGGGTCCGAACTGTGAAATACGAAGCCGAGCATATTACTCACCTTAACACTCTCGCCTATTGTTAAAG 5′

-7T/A (premelted)

5′ CAGTGAGCGCAACGCAATAAATGTGATCTAGATCACATTTTAGGCACCCCAGGCTTGACACTTTATGCTTCGGCTCGTATAAAGTGTGGAATTGTGAGAGCGGATAACAATTTC 3′
3′ GTCACTCGCGTTGCGTTATTTACACTAGATCTAGTGTAAAATCCGTGGGGTCCGAACTGTGAAATACGAAGCCGAGCATCGGCACAACCTTAACACTCTCGCCTATTGTTAAAG 5′

Nucleotides conditions and RNA transcripts produced in experiments:
ITC6: ApA and 80 μM UTP/GTP; **ITC7:** ApA and 80 μM UTP/GTP; **ITC11:** ATP (or ApA) and (ATP/)GTP/UTP
ITC6: 5′-AAUUGU-3′; **ITC7:** 5′-AAUUGUG-3′; **ITC11:** 5′-AAUUGUGAGAG-3′

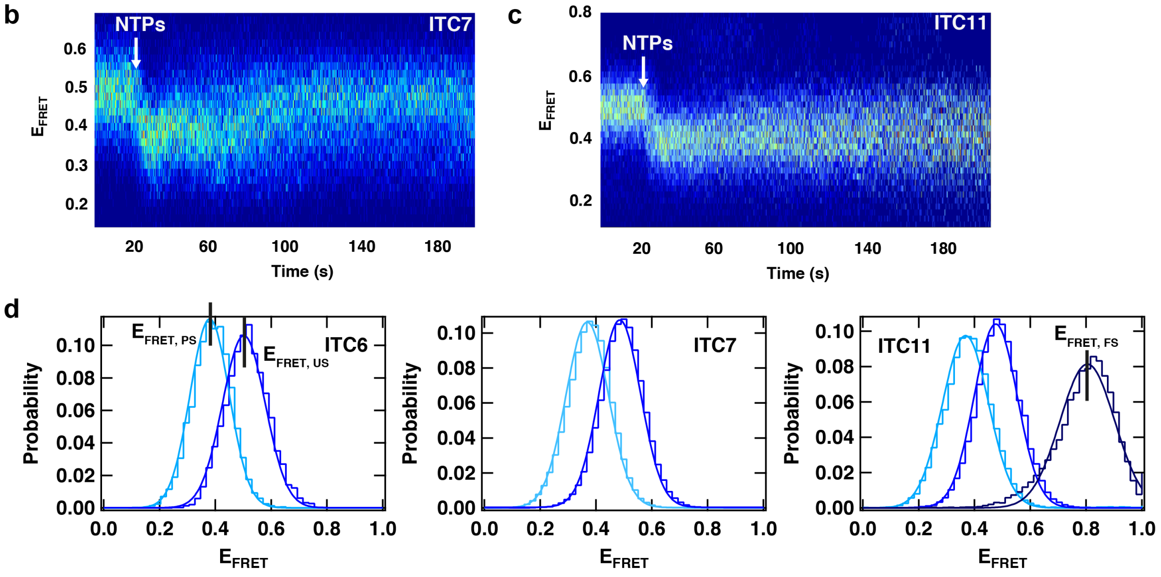

e

	$E_{FRET, US}$ (*)	$E_{FRET, PS}$ (**)	$E_{FRET, FS}$ (***)
ITC6	0.50	0.38	-
ITC7	0.49	0.37	-
ITC11	0.48	0.37	0.80

(*) US: unscrunched; (**) PS: partly scrunched; (***) FS: fully scrunched.

Supplementary Figure 1: DNA promoter sequences and initial transcribing single-molecule FRET calibration. (**a**) The different DNA promoter sequences used in the study. The arrow indicate the start site and the light blue font indicates the premelted region. **(b)** Transcription heatmap in ITC7 condition (500 μM ApA and 80 μM GTP-UTP) using the WT DNA construct. **(c)** Transcription heatmap in ITC11 condition (500 μM ApA and 80 μM GTP/UTP/ATP) using the WT DNA construct. **(d)** Histograms reporting the FRET efficiency E_{FRET} for each FRET level, i.e. low, intermediate and high represented in light blue, blue and dark blue respectively, in each ITC conditions, i.e. ITC6, ITC7 and ITC11. The solid lines are Gaussian fits that provide the center of each distribution. **(e)** FRET levels center extracted from the Gaussian fit performed in (d) for the FRET levels of the US, PS and FS states for each Initial Transcribing Complex (ITC).

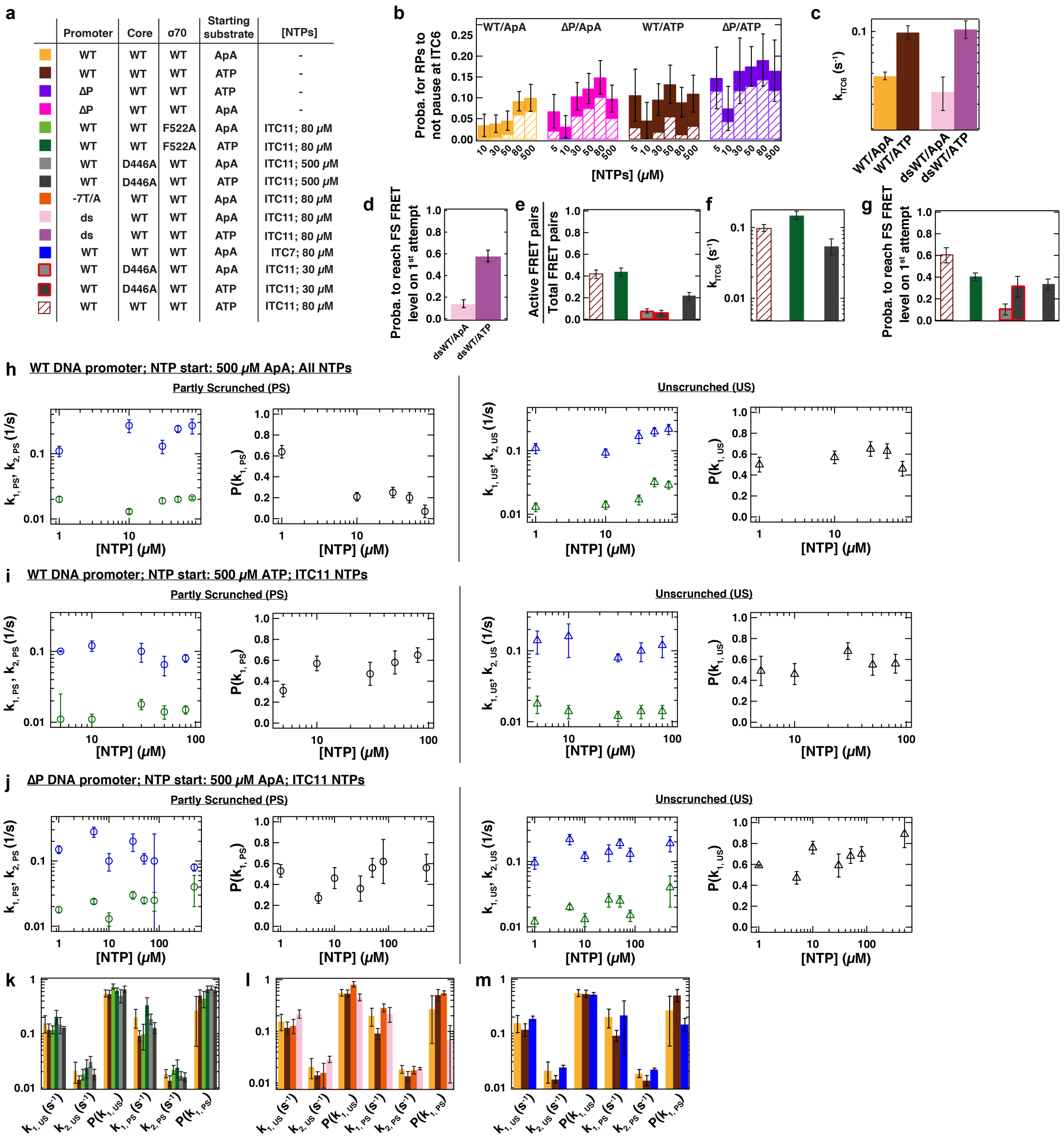

Supplementary Figure 2: Kinetics of the ITC6 pause and the US and PS FRET states for different NTPs concentrations, different initiating nucleotides, dsWT promoter, and core and σ[70] mutants. Starting substrate is either ApA or ATP, at 500 μM concentration. **(a)** Table with the different experimental variables explored in (b)-(j) and the color code associated. Data represented in yellow and brown in (h, i, j) are averaged values for all NTP concentrations (see **Figure 2a**), and in (c) the individual values for each concentration of NTPs (b). **(b)** Probability to reach the FS FRET level without pausing at ITC6. The dashed bars are the data corrected from the fraction of the ITC6 pause exponential distribution that is faster than our time limit (the frame time, i.e. 80 ms for ΔP and ATP and 200 ms otherwise, times two as we keep pauses longer or equal to two frames). The data are extracted from **Supplementary Table 1**. The error bars are values at 95% confidence interval. **(c)** Exit rate k_{ITC6} (**Figure 1d**) using the WT DNA promoter sequence or the dsWT promoter (**Supplementary Figure 1a**) as a function of NTP start. Data from **Figure 2d** (yellow and brown) are represented here for comparison. **(d)** Probability to escape the ITC6 pause on the first attempt as in **Figure 1d** for the conditions in (c). **(e)** Ratio of the number of FRET pairs, i.e. a pair of made of a single acceptor dye and a single donor dye that demonstrate FRET (see **Materials and Methods: FRET pair localization and detection**), showing transcription activity over the total number of FRET pairs detected for the experimental

conditions described in (a). **(f)** ITC6 pause exit rate for the experimental conditions described in (a). **(g)** Probability to reach the FS FRET level on the 1st attempt for the experimental conditions described in (a). **(h, i, j)** Parameters from a two-exponential Maximum Likelihood Estimation (MLE) fit of the distribution of the dwell times from the US and PS FRET levels from the complexes that did not reach the FS FRET level on the first attempt (**Figure 1d**) as a function of the concentration of NTPs: exit rates $k_{1,\,PS}$ (blue circles), $k_{2,\,PS}$ (green circles) and $P(k_{1,\,PS})$ the probability to exit with $k_{1,\,PS}$ for the PS FRET level; exit rates $k_{1,\,US}$ (blue triangles), $k_{2,\,US}$ (green triangles) and $P(k_{1,\,US})$ the probability to exit with $k_{1,\,US}$ for the US FRET level. **(k, l, m)** Parameters extracted from a double-exponential MLE fitting of the US and PS FRET levels as described in (h, i, j) for the experimental conditions described in (a).

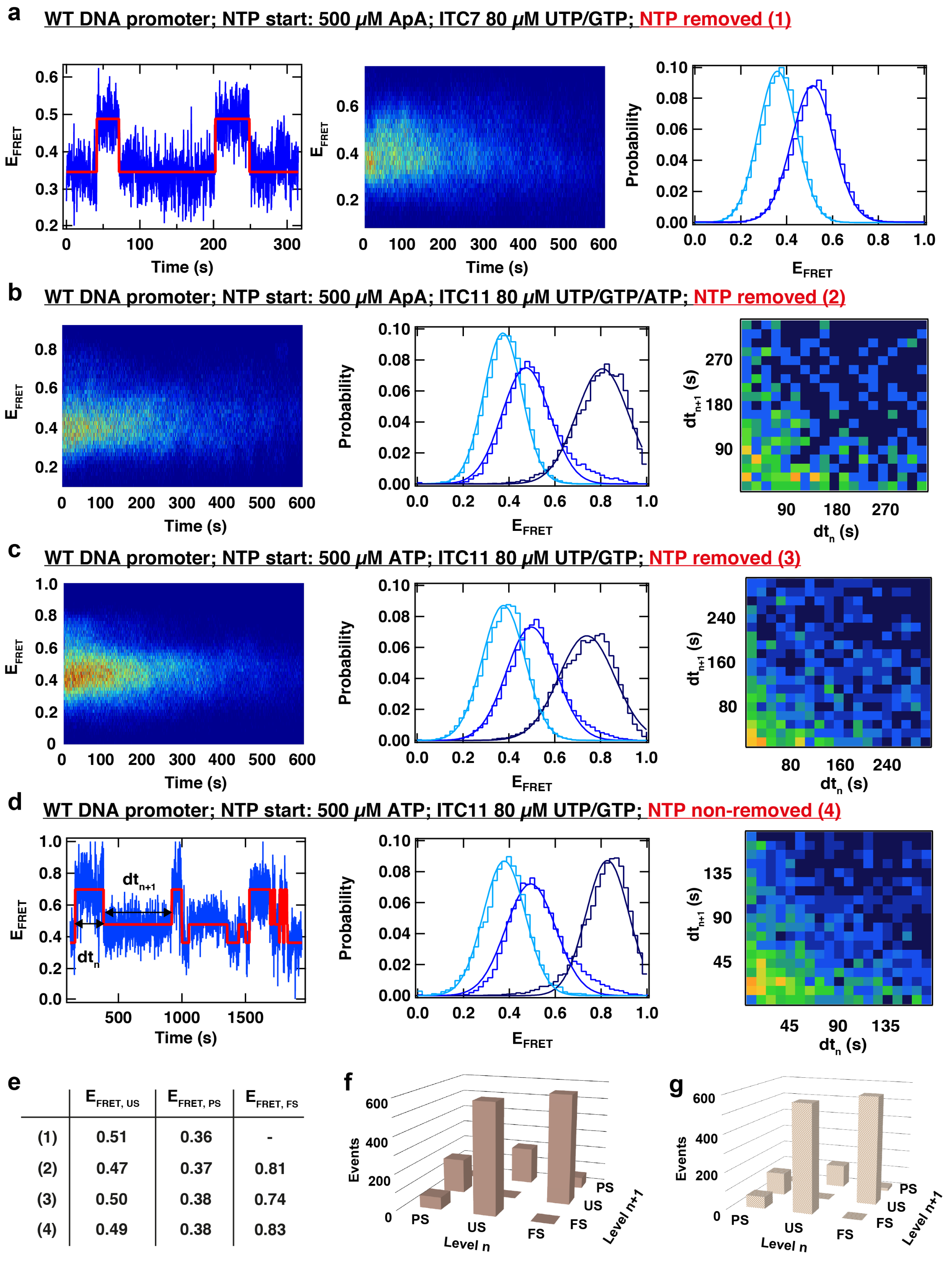

	$E_{FRET, US}$	$E_{FRET, PS}$	$E_{FRET, FS}$
(1)	0.51	0.36	-
(2)	0.47	0.37	0.81
(3)	0.50	0.38	0.74
(4)	0.49	0.38	0.83

Supplementary Figure 3: Halted RP_{ITC} show similar scrunching activity in the presence or the absence of NTPs. The experiments here are performed with the WT DNA promoter sequence (**Supplementary Figure 1a**). **(a)** FRET trace of transcription complex that has been incubated in ITC7 conditions followed by NTP removal as described in **Figure 4a**, transcription heatmap and histogram of the PS (light blue) and US (blue) FRET levels. **(b)** and **(c)** transcription complexes alternate between US, PS and FS FRET levels after NTP removal after incubation in ITC11 condition (**Figure 4a**) with either ApA (b) or ATP (c) as starting substrate. Here is represented a transcription heatmap, the FRET levels histograms and their respective Gaussian

fits (solid line) and a correlation map. The correlation map represents the duration of the dwell time dt_n as a function of the duration of the subsequent dwell time dt_{n+1} (time trace in (d)). **(d)** As in (b) and (c) but in the presence of NTPs (ITC11 condition, ATP starting substrate and 80 μM NTPs) and after reaching the FS FRET level on the first attempt. The FRET levels histograms and their respective Gaussian fits (solid line) are represented in the second panel and the correlation map of two by two successive dwell times dt, as in (b) and (c). **(e)** FRET levels average values extracted from the peak position of the Gaussian fits performed in (a), (b), (c) and (d) for the US, PS and FS FRET levels. **(e, g)** 3D histogram showing the number of transitions occurring between the different FRET levels for the n and n+1 successive dt, for the experiments described in (c) and (d).

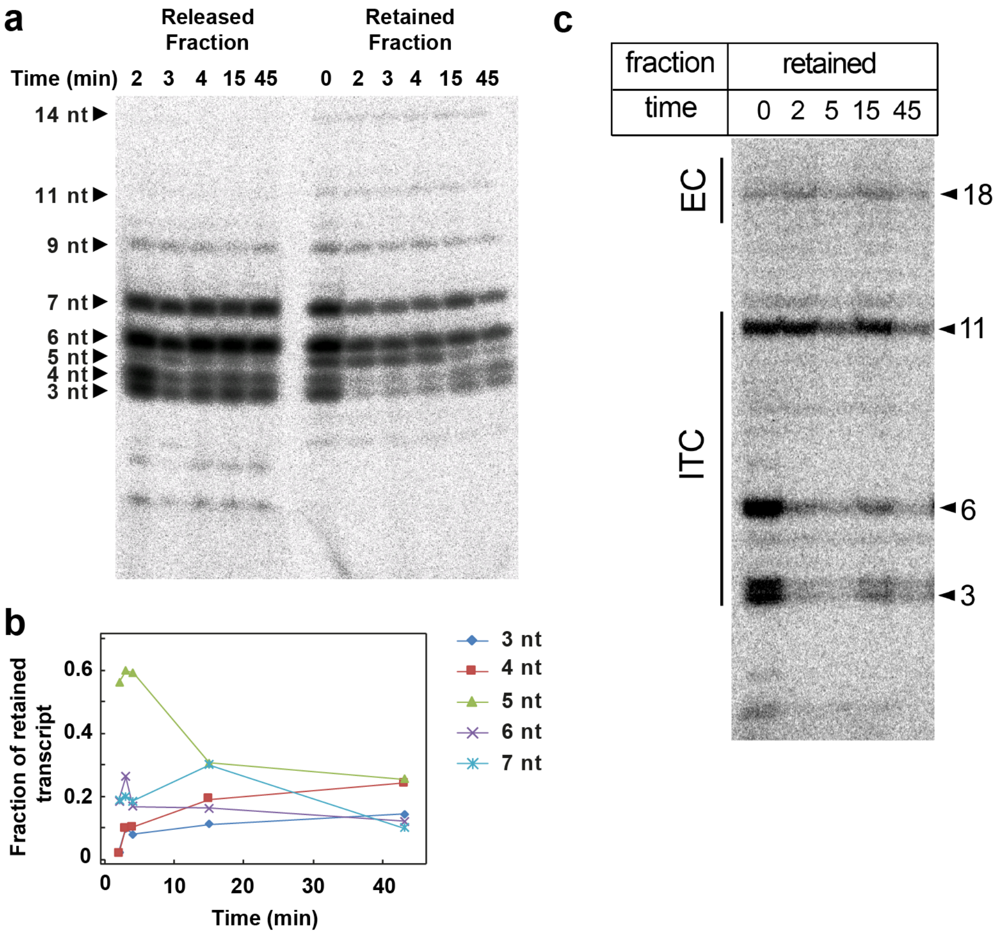

Supplementary Figure 4: Bead-attached ITCs forms a stable complex with the nascent RNA that is competent for elongation. (a) Gel electrophorisis assay showing the part of the nascent RNA trancripts that is released and retained as a function of the waiting time after NTP removal (ITC7 conditions, see **Supplementary Methods**). The indicated duration is the time from the start of the experiments. **(b)** Analysis of the gel from (a) showing the fraction of the retained transcript as a function of time and length of the transcript. The apparent increase in the fraction of retained 3- and 4-nt RNA between 15 and 45 min is explained by the cleavage of the 5 nt RNA by the nuclease activity of the RNAP. **(c)** Gel electrophorisis assay similar to (a) in ITC11 conditions (**Supplementary Methods**).

References

1 Kulbachinskiy, A. & Mustaev, A. Region 3.2 of the sigma subunit contributes to the binding of the 3'-initiating nucleotide in the RNA polymerase active center and facilitates promoter clearance during initiation. *The Journal of biological chemistry* **281**, 18273-18276, doi:10.1074/jbc.C600060200 (2006).

2 Duchi, D. *et al.* RNA Polymerase Pausing during Initial Transcription. *Molecular Cell* **63**, 939-950, doi:10.1016/j.molcel.2016.08.011 (2016).

3 Bauer, D. L. V., Duchi, D. & Kapanidis, A. N. E. Coli RNA Polymerase Pauses during Initial Transcription. *Biophysical journal* **110**, 21a-21a (2016).

4 Lerner, E. *et al.* Backtracked and paused transcription initiation intermediate of Escherichia coli RNA polymerase. *Proceedings of the National Academy of Sciences of the United States of America* **113**, E6562-E6571, doi:10.1073/pnas.1605038113 (2016).

5 Revyakin, A., Allemand, J. F., Croquette, V., Ebright, R. H. & Strick, T. R. Single-molecule DNA nanomanipulation: detection of promoter-unwinding events by RNA polymerase. *Methods in enzymology* **370**, 577-598, doi:10.1016/S0076-6879(03)70049-4
S0076687903700494 [pii] (2003).

6 Revyakin, A., Ebright, R. H. & Strick, T. R. Promoter unwinding and promoter clearance by RNA polymerase: detection by single-molecule DNA nanomanipulation. *Proceedings of the National Academy of Sciences of the United States of America* **101**, 4776-4780, doi:10.1073/pnas.0307241101
0307241101 [pii] (2004).

7 Revyakin, A., Liu, C., Ebright, R. H. & Strick, T. R. Abortive initiation and productive initiation by RNA polymerase involve DNA scrunching. *Science (New York, N.Y* **314**, 1139-1143, doi:314/5802/1139 [pii]
10.1126/science.1131398 (2006).

8 Marko, J. F. Torque and dynamics of linking number relaxation in stretched supercoiled DNA. *Physical review. E, Statistical, nonlinear, and soft matter physics* **76**, 021926, doi:10.1103/PhysRevE.76.021926 (2007).

9 Brutzer, H., Luzzietti, N., Klaue, D. & Seidel, R. Energetics at the DNA supercoiling transition. *Biophysical journal* **98**, 1267-1276, doi:10.1016/j.bpj.2009.12.4292 (2010).

10 Janssen, X. J. A. *et al.* Electromagnetic Torque Tweezers: A Versatile Approach for Measurement of Single-Molecule Twist and Torque. *Nano letters* **12**, 3634-3639, doi:Doi 10.1021/Nl301330h (2012).

11 Strick, T. R. *et al.* Stretching of macromolecules and proteins. *Reports on Progress in Physics* **66**, 1-45 (2003).

12 Toulme, F. *et al.* GreA and GreB proteins revive backtracked RNA polymerase in vivo by promoting transcript trimming. *The EMBO journal* **19**, 6853-6859, doi:10.1093/emboj/19.24.6853 (2000).

13 Henderson, K. L. *et al.* Mechanism of transcription initiation and promoter escape by E. coli RNA polymerase. *Proceedings of the National Academy of Sciences of the United States of America* **114**, E3032-E3040, doi:10.1073/pnas.1618675114 (2017).

14 Susa, M., Kubori, T. & Shimamoto, N. A pathway branching in transcription initiation in Escherichia coli. *Molecular microbiology* **59**, 1807-1817, doi:10.1111/j.1365-2958.2006.05058.x (2006).

15 Kubori, T. & Shimamoto, N. A branched pathway in the early stage of transcription by Escherichia coli RNA polymerase. *Journal of molecular biology* **256**, 449-457, doi:10.1006/jmbi.1996.0100 (1996).

16 Strobel, E. J. & Roberts, J. W. Two transcription pause elements underlie a sigma70-dependent pause cycle. *Proceedings of the National Academy of Sciences of the United States of America* **112**, E4374-4380, doi:10.1073/pnas.1512986112 (2015).
